# Supplementary figures and images for: MLL methyltransferases regulate H3K4 methylation to ensure CENP-A assembly at human centromeres
Source: PLoS Biol. 2023 Jun 28;21(6):e3002161. doi: 10.1371/journal.pbio.3002161 (PMC10335677; doi:10.1371/journal.pbio.3002161)

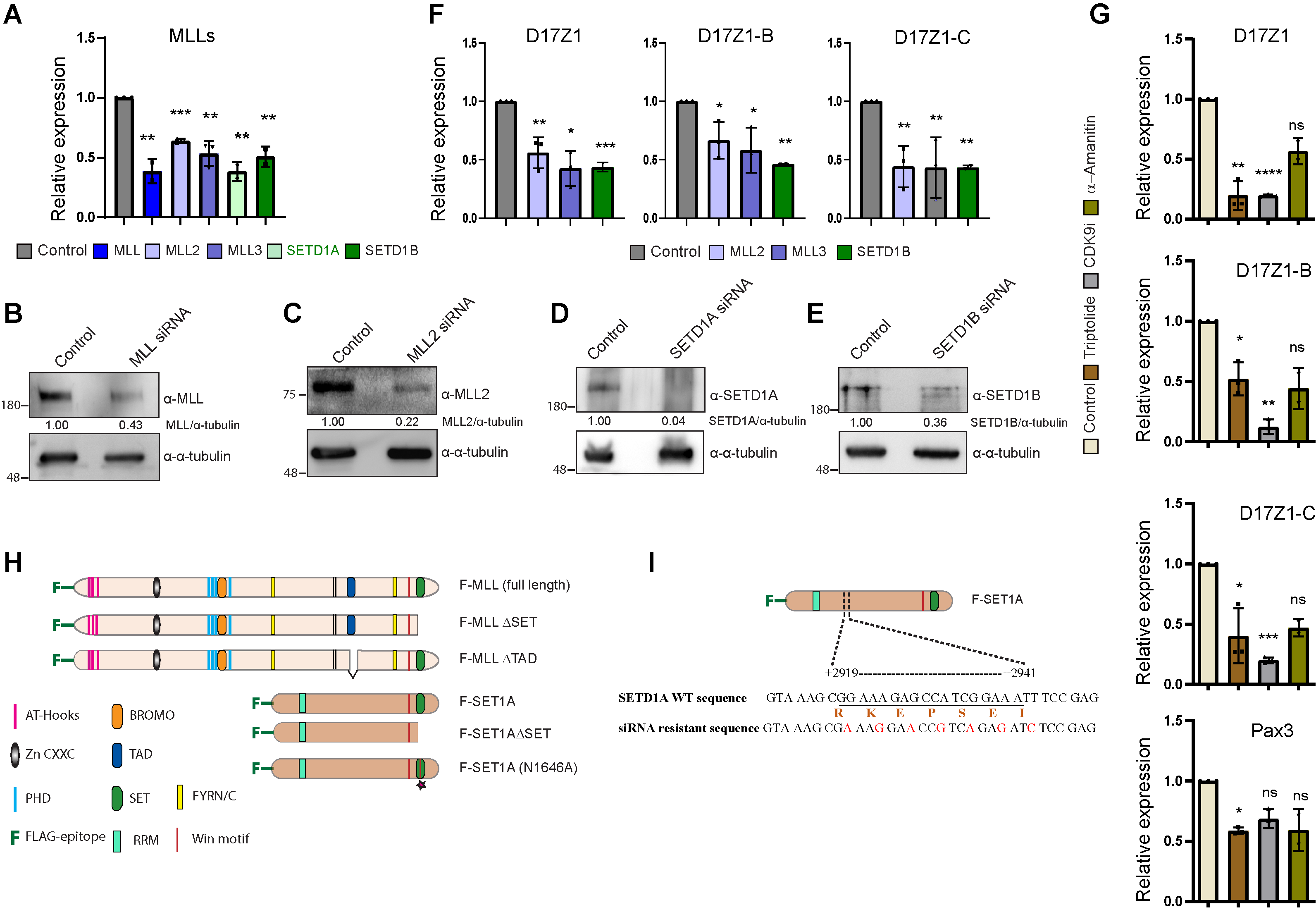

Supplement: S1 Fig — (A) siRNA-mediated down-regulation of various MLLs was performed and the efficacy of siRNA treatment was determined by plotting the qRT-PCR analysis of respective transcript levels after total RNA extraction and cDNA synthesis. (B–E) Immunoblots of whole-cell lysate were prepared from cells treated with either control and MLL (B), MLL2 (C), SETD1A (D), or SETD1B (E) siRNA to analyze the respective protein levels. Blots were probed with α-MLLC (B), α-MLL2 (C), α-SETD1A (D), and α-SETD1B (E) and α-tubulin as shown. Molecular weight markers (in kDa) and relative quantification are shown as indicated. Uncropped blots provided in S1 Raw Images. (F) cDNA samples obtained after RNAi treatment of Control, MLL2, MLL3, and SETD1B (from A) were analyzed for cenRNA transcripts from D17Z1, D17Z1-B, and D17Z1-C α-satellite arrays of chromosome 17. (G) qRT-PCR analysis of α-satellite cenRNA from individual HOR of chromosome 17 and RNA Pol II regulated gene PAX3, after treatment with either control (DMSO), Triptolide (20 μm), CDK9 inhibitor (20 μm), α-amanitin (20 μg) for 4 h, is shown. (A, F, G) cDNA was synthesized from total RNA after rigorous DNase I treatment and amplified using qRT-PCR for indicated RNAs. Data from all samples were normalized to GAPDH mRNA levels from respective samples by using − ΔΔCT method and expression is shown relative to control siRNA-treated/DMSO-treated cells from respective cell line/treatment (which is arbitrarily set to 1). Each experiment was performed at least 3, or more times except α-amanitin treatment (2 times). Error bars represent SD. *P ≤ 0.05, **P ≤ 0.005, ***P ≤ 0.0005, ****P ≤ 0.0001, ns: not significant, P > 0.05 (two-tailed Student’s t test). (H) Schematic representation of recombinant MLL and SETD1A FLAG-epitope tagged mutants used in this study displaying different domains in these proteins. Full-length MLL (FL) and SET domain deleted MLL (Δaa3829–3969) U-2OS cell lines have been described before [35]. MLLΔTAD was generated in full- [file pbio.3002161.s001.tif]

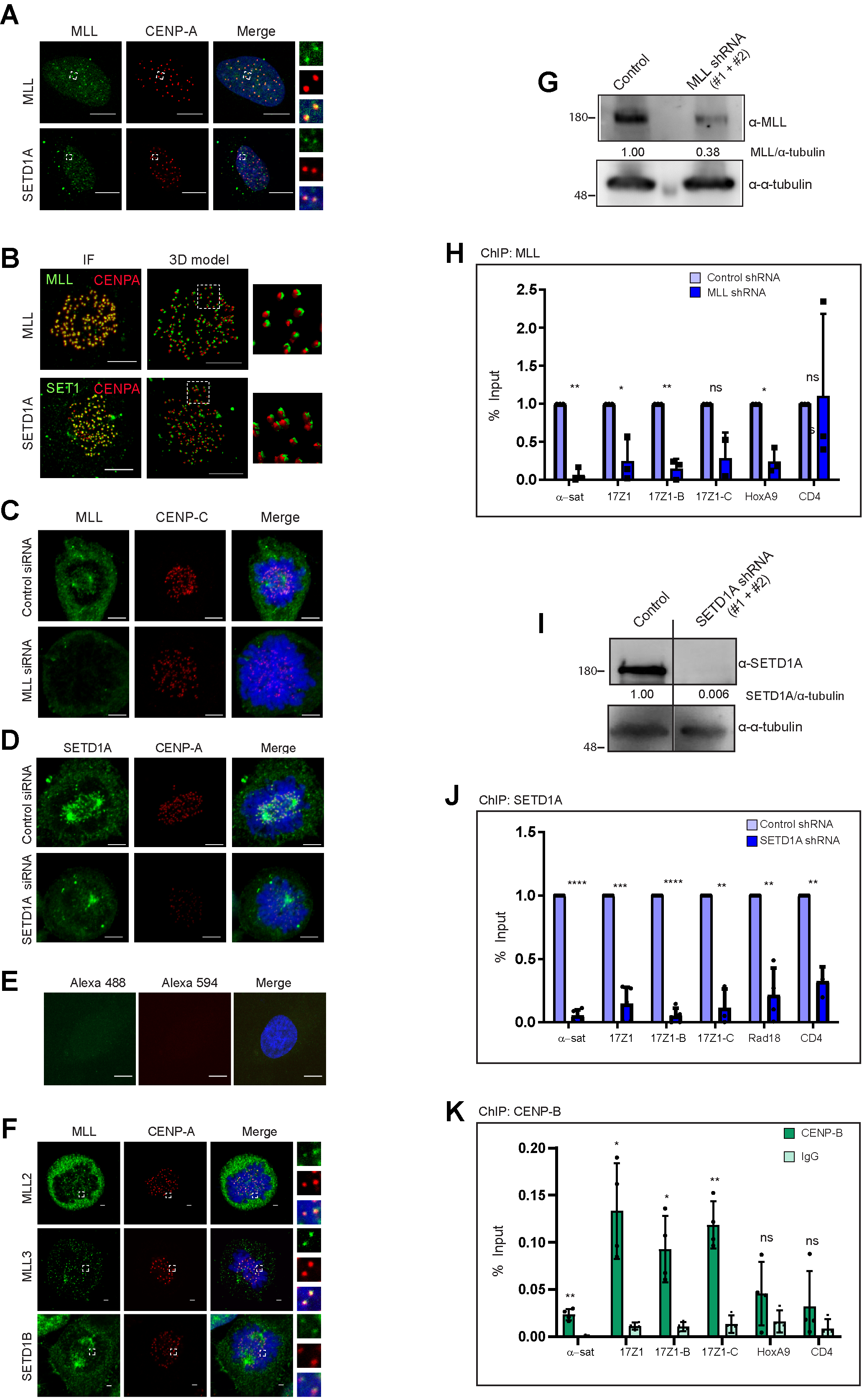

Supplement: S2 Fig — (A) Immunofluorescence staining (IF) of endogenous MLL (green) or SETD1A (green) with CENP-A (red) in U-2OS cells in interphase is shown. DNA was stained with DAPI (blue). The area in the white square is magnified and shown on the right for each image. (B) A 3D view of endogenous MLL (green) upper panel or SETD1A (green) lower panel. with CENP-A (red), is shown. The magnified area of the 3D model is shown on the right. (C-D) U-2OS cells were transfected with Control, MLL (C), or SETD1A (D) siRNA to check for the specificity of MLL or SETD1A staining at the centromere. Cells were stained with endogenous MLL (green) or SETD1A (green) with CENP-C (red) or CENP-A (red), and DNA was stained with DAPI (blue) as indicated. (E) U-2OS cells, stained with Alexa Flour 488 and Alexa Flour 594, are shown. (A–E) Scale bar, 5 μm. (F) U-2OS cells were stained with MLL2 (green), MLL3 (green), or SETD1B(green) antibody along with centromeric marker CENP-A (red) as shown. The area in the white square is magnified and shown on the left for each image. Scale bar, 2 μm. (G, I) Immunoblot show MLL (G) and SETD1A (I) shRNA (#1 and #2) knockdown efficiency in treated HEK-293 cells. The blots were probed with α-MLL (G) or α-SETD1A (I), and α-α-tubulin antibody. Uncropped blots provided in S1 Raw Images. (H, J) Chromatin immunoprecipitation (ChIP) analyzes showing decrease in levels of MLL (H) and SETD1A (J) at centromeric α-satellite loci following treatment of either MLL shRNA or SETD1A shRNA in HEK-293 cells, and the result plotted as percent input enrichment, are shown. Each experiment was performed at least 3 or more times. Error bars represent SD. *P ≤ 0.05, **P ≤ 0.005, ***P ≤ 0.0005, ****P ≤ 0.0001, ns: not significant, P > 0.05 (two-tailed Student’s t test). (K) CENP-B occupancy, detected by ChIP, is shown. Data from 3 or more independent ChIP experiments is shown. Error bars represent SD. *P ≤ 0.05, **P ≤ 0.005, ns: not significant, P > 0.05 (two-way ANOVA with Šídák multiple compar [file pbio.3002161.s002.tif]

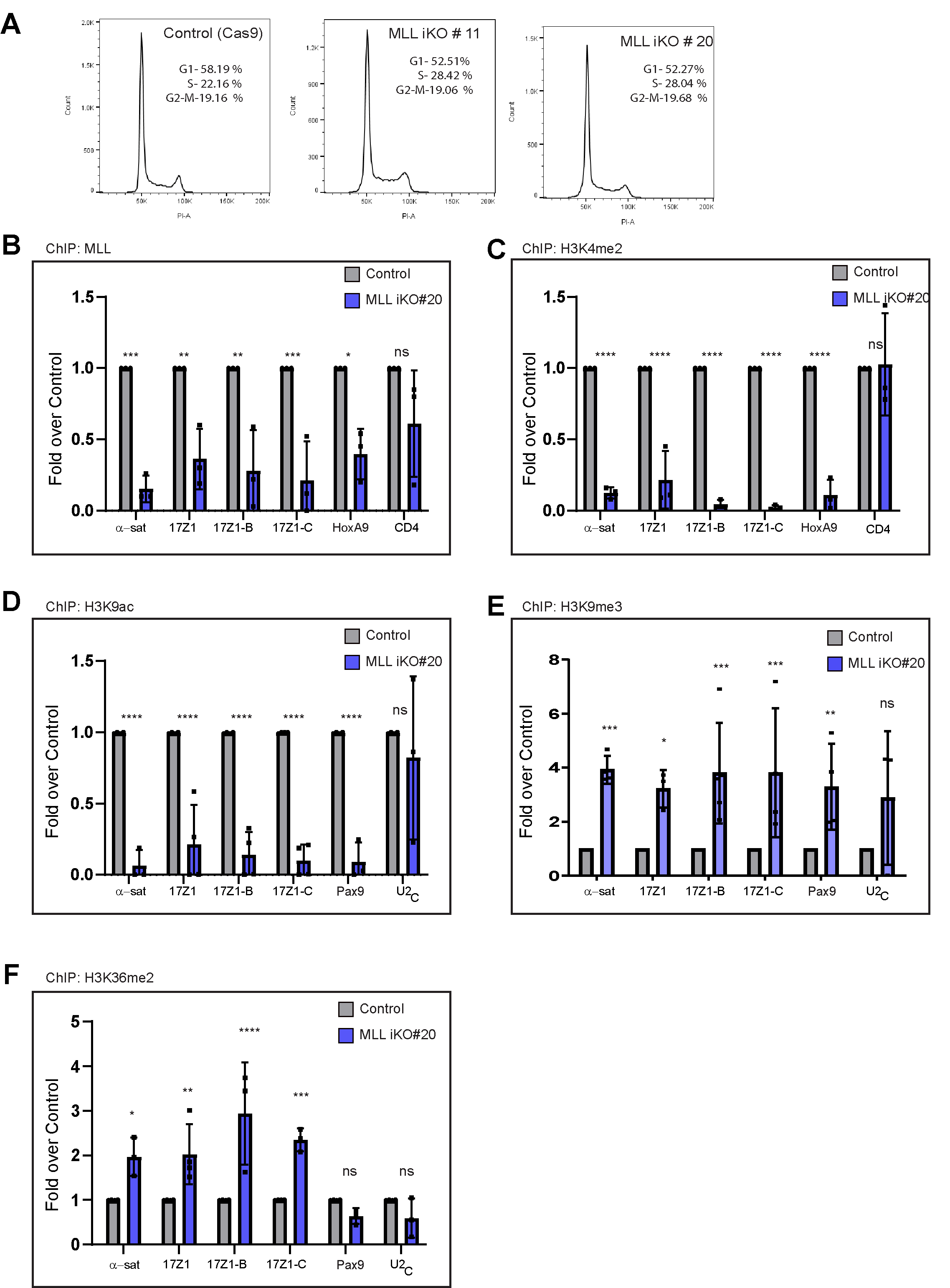

Supplement: S3 Fig — (A) Cell cycle profile obtained from flow cytometry analysis of MLL KO (iKO #11 and #20) cells after 7 days of Doxycycline treatment is shown. Representative graph (from 3 independent experiments) is shown with values obtained for sample shown here. Parental (or Cas9-expressing) cells are used as Control. The raw data underlying this part can be found here (http://flowrepository.org/id/FR-FCM-Z6AH). (B–F) ChIP-analyzes with MLL (B), H3K4me2 (C), H3K9ac (D), H3K9me3 (E), and H3K36me2 (F) antibodies in MLL iKO cells (#20) are shown. Data were normalized against the ChIP values obtained in parental (or Cas9-expressing) cells, which are used as Control. Data from 3 or more independent ChIP experiments are plotted. Error bars represent SD. *P ≤ 0.05, **P ≤ 0.005, ***P ≤ 0.0005, ****P ≤ 0.0001, ns: not significant, P > 0.05 (two-way ANOVA with Šídák multiple comparison test). α-sat, α-satellite. The raw data underlying parts (B–F) can be found in S1 Data. (TIF) [file pbio.3002161.s003.tif]

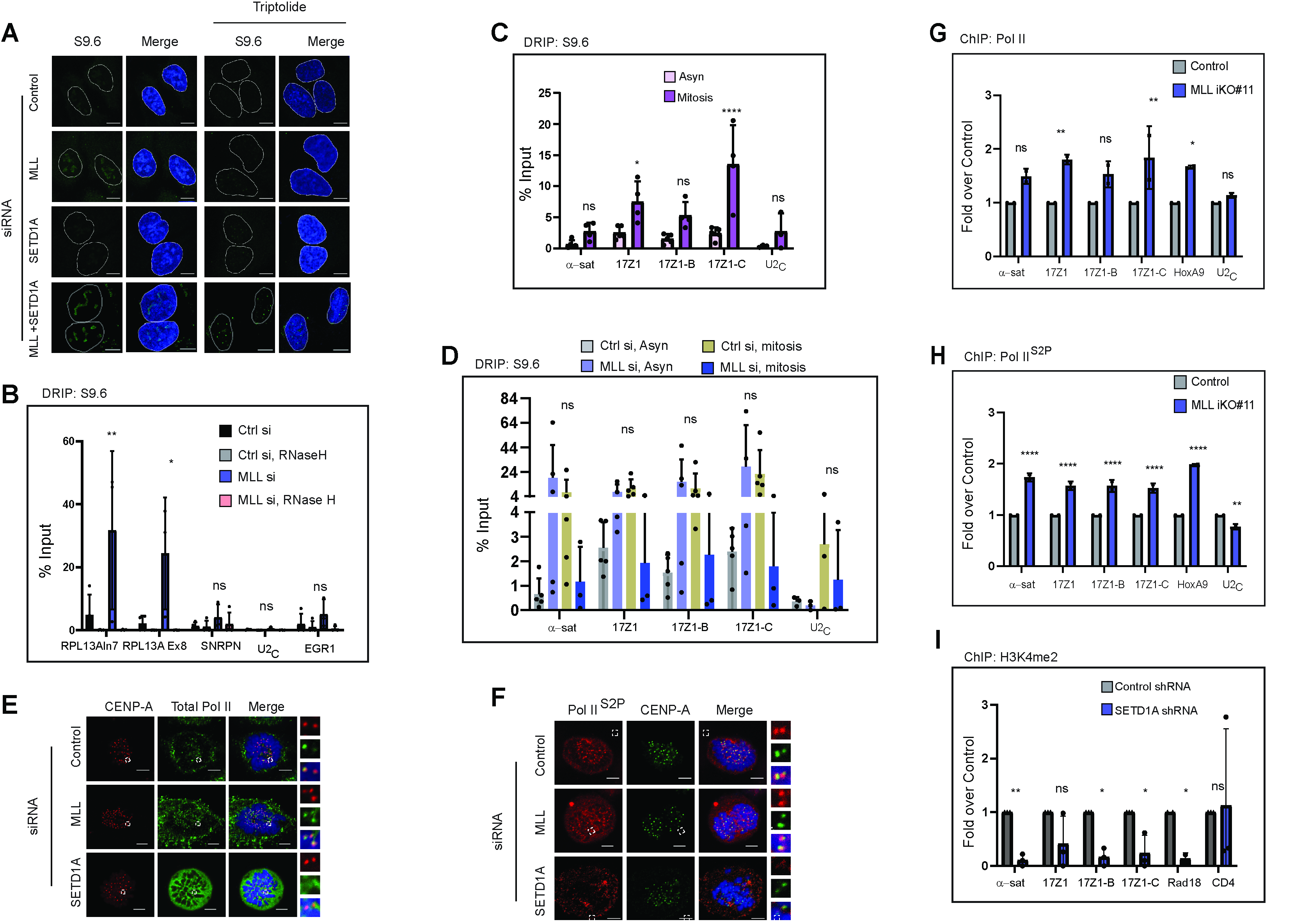

Supplement: S4 Fig — (A) Representative images for the data represented in Fig 4A show nuclear R-loops, 48 h after MLL and SETD1A and MLL+SETD1A siRNA treatment in U-2OS cells. The cells were stained using the S9.6 (green) antibody and DAPI (blue). Each nucleus is outlined in white. For transcription inhibition, cells were treated with 20 μm Triptolide or DMSO (Control) for 4 h. Scale bar, 10 μm. (B) DRIP analysis in MLL RNAi-treated HEK-293 cells, with respective RNase H controls, is shown. Data are presented as percent input enrichment. Data from 3 or more independent DRIP experiments are plotted. (C, D) DRIP analysis in HEK-293 cells, with asynchronous or cells synchronized in mitosis, is shown. Data are presented as percent input enrichment. Data from 3 or more independent DRIP experiments are plotted. Data from D is replotted in C for Control cells to highlight the changes observed in mitosis. Error bars represent SD. **P ≤ 0.005, ***P ≤ 0.0005, ns: not significant, P > 0.05 (two-way ANOVA with Šídák multiple comparison test). (E, F) Immunofluorescence staining of Total RNA Pol II (E, green) or RNA Pol IIS2P (F, green) and CENP-A (red) in mitotic cells following treatment with either Control, MLL, or SETD1A siRNA, are shown. Scale bar, 5 μm. (G–I) ChIP-analysis of RNA Pol II (G) and RNA Pol IIS2P (H) in MLL knock out (iKO #11) and H3K4me2 (I) in SETD1A shRNA treated HEK-293 cells is shown. Data were normalized against the ChIP values obtained in Control samples and presented as fold change over control. Data from 3 or more independent experiments were plotted. Error bars represent SD. *P ≤ 0.05, **P ≤ 0.005, ****P ≤ 0.0001, ns: not significant, P > 0.05 (two-way ANOVA with Šídák multiple comparison test). Ctrl, control; si, siRNA; Asyn, asynchronous. The raw data underlying parts (B–D) and (G–I) can be found in S1 Data. (TIF) [file pbio.3002161.s004.tif]

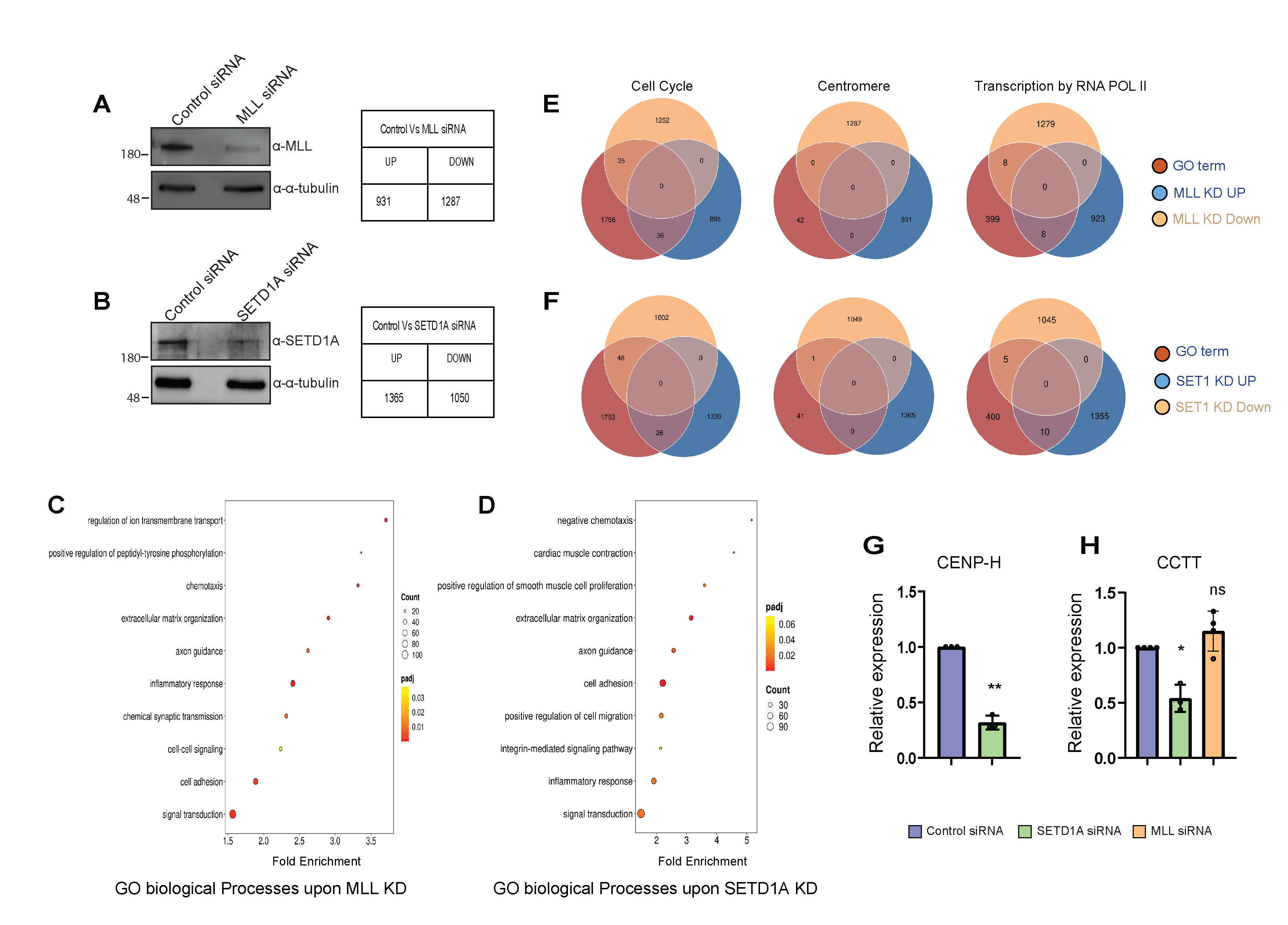

Supplement: S5 Fig — (A, B) U-2OS cells were either treated with Control, MLL (A), or SETD1A (B) siRNA, and immunoblots of whole-cell lysate showing MLL (A), SETD1A (B), and respective tubulin level are shown. Uncropped blots provided in S1 Raw Images. Number of genes up- or down-regulated significantly (log2FC > 1 or log2FC < −1 and padj < 0.05, Benjamini–Hochberg method) in MLL (A) or SETD1A (B) siRNA-treated cells were listed (also see S1 Table). (C, D) GO enrichment analysis of differentially regulated transcripts were identified in MLL (C) and SETD1A (D) siRNA-treated cells. Biological processes of GO terms were ranked based on the adjusted p-value obtained from online web server DAVID. Ten most significant enriched GO terms are presented. The color represents padj value and the diameter of the circle size indicates no of genes in that category. (E, F) Venn diagram showing the overlap between the gene ontology annotations related to cell cycle, centromere, and transcription by RNA Pol II among DEGs in MLL-KD (E) and SETD1A-KD (F) (also see S2 and S3 Tables). (G) SETD1A siRNA-treated cDNA samples were analyzed for CENP-H transcript levels as shown. (H) cDNA samples obtained after RNAi treatment of Control, SETD1A, and MLL, were analyzed for CCTT lncRNA transcripts levels. (G, H) *P ≤ 0.05, **P ≤ 0.005, ns: not significant, P > 0.05 (two-tailed Student’s t test). SET1, SETD1A; up/down, up-regulated/down-regulated genes; GO, Gene Ontology; DEGs, differentially expressed genes; CCTT, CENP-C targeting transcript. The raw data underlying parts (G, H) can be found in S1 Data. (TIF) [file pbio.3002161.s005.tif]

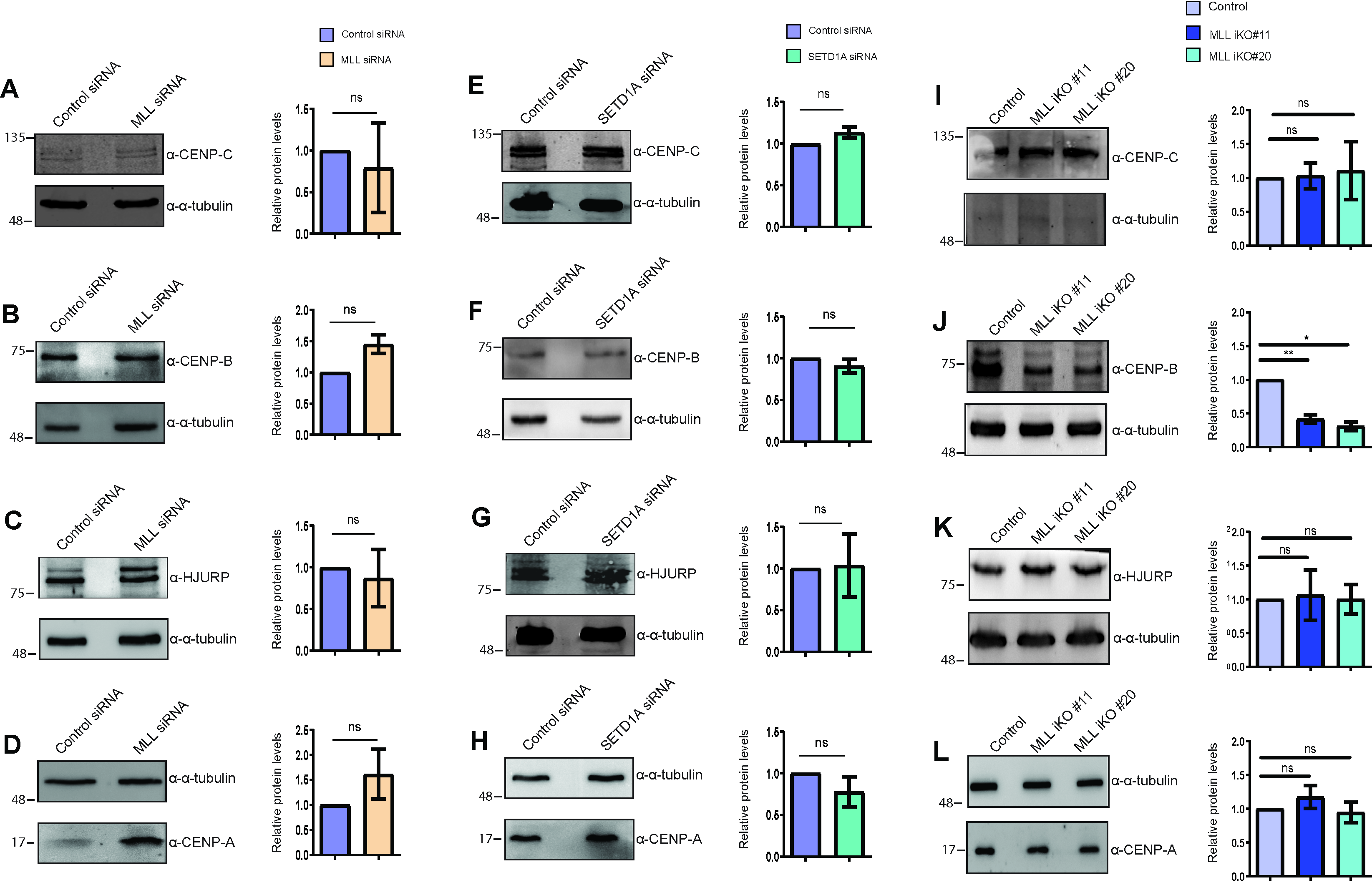

Supplement: S6 Fig — (A–D) Immunoblot shows CENP-C (A), CENP-B (B), HJURP (C), and CENP-A (D) protein levels in Control or MLL siRNA-treated U-2OS cells. (E–H) Immunoblot shows CENP-C (E), CENP-B (F), HJURP (G), and CENP-A (H) protein levels in Control or SETD1A siRNA-treated U2OS cells. (I–L) Immunoblot analysis of CENP-C (I), CENP-B (J), HJURP (K), and CENP-A (L) protein levels in MLL HEK-293 KOs (iKO #11 and #20) cell lines are shown. Blots were probed with respective antibodies as indicated. Molecular weight markers (in kDa) are shown on the left and quantitative analysis of respective protein levels from 2 independent experiments is shown on the right. Each bar represents mean SD. **P ≤ 0.005, ns: not significant, P > 0.05. (A–H, two-tailed Student’s t test; I–L, two-way ANOVA with Šídák multiple comparison test). Uncropped blots provided in S1 Raw Images. The raw data underlying parts (A–L) can be found in S1 Data. (TIF) [file pbio.3002161.s006.tif]

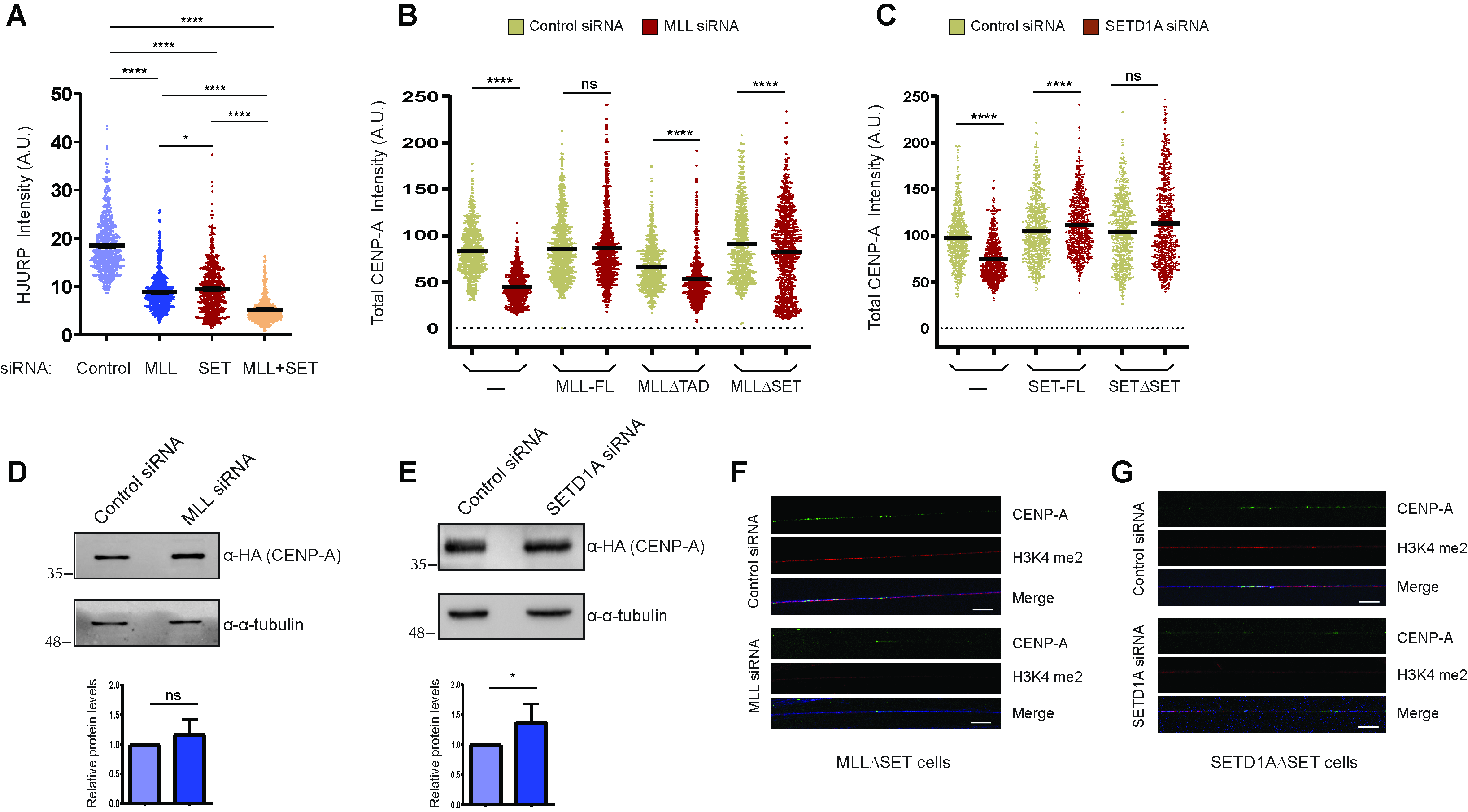

Supplement: S7 Fig — (A) Quantification of HJURP fluorescence intensity following depletion of Control, MLL, SETD1A, or MLL+SETD1A. Each data point represents a single centromere. Quantification of HJURP fluorescence images shown in Fig 6G) The error bar represents SEM ≥250 centromeres quantified from 10 early G1 cell pairs, (n = 3 experiments). ****P ≤ 0.0001, *P ≤ 0.05, ns: not significant, P > 0.05 (Ordinary one-way ANOVA with Tukey’s multiple comparisons test). (B) Quantification of centromeric fluorescence intensity of ectopically expressed total CENP-A (stained using α-HA antibody) in parent U-2OS cells (—) or cell line stably expressing MLL full length (FL) or MLLΔTAD or MLLΔSET upon MLL siRNA treatment. (C) Quantification of centromeric fluorescence intensity of total CENP-A in parent U-2OS cells (—) or cell line stably expressing siRNA resistant SETD1A full length (FL) or SETD1AΔSET (here, N1646A mutant was used) upon SETD1A siRNA. Each data point represents a single centromere; n ≥ 300 quantified from 10 early G1 cell pairs, (n = 2 experiments). ****P ≤ 0.001, ns: not significant, P > 0.05 (Mann–Whitney two-tailed unpaired test). (D, E) Stably expressing CENP-A SNAP-3xHA cells were either transfected with Control, MLL (D), or SETD1A (E) siRNA, and collected after 72 h for whole-cell lysate preparation. CENP-A-SNAP-3xHA protein was detected using an α-HA antibody. Uncropped blots provided in S1 Raw Images. Quantitative analysis of relative total protein levels are shown (lower panel). Each bar represents mean SD of 2 biological replicates. *P ≤ 0.05, ns: not significant. (F, G) Immunofluorescence staining (IF) of chromatin fiber prepared from cell line stably expressing MLLΔSET (F) or SETD1AΔSET (G, here N1646A mutant was used). Stably cell line cells were transfected either with Control, MLL siRNA (F), or SETD1A (G) siRNA and the chromatin fibers were extracted and stained with endogenous CENP-A (green) and H3K4me3 (red), and DNA stained with DAPI (blue). (F) Frequency of H3K4 [file pbio.3002161.s007.tif]

3-A

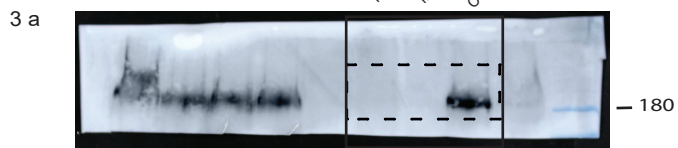

3 b

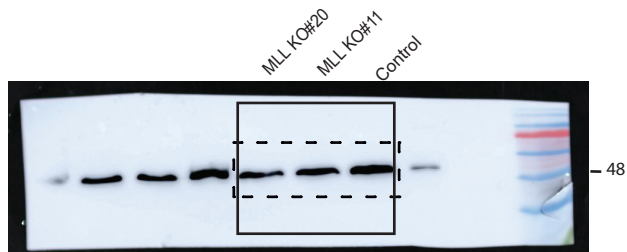

S1-B

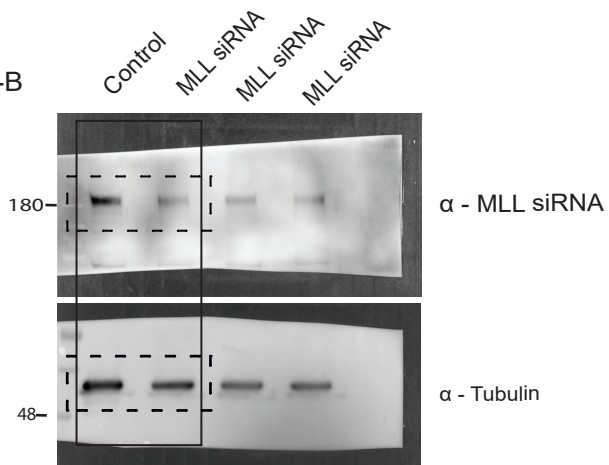

S1-C

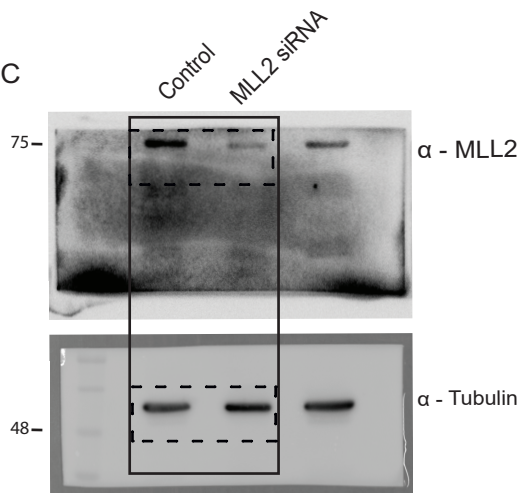

S1-D

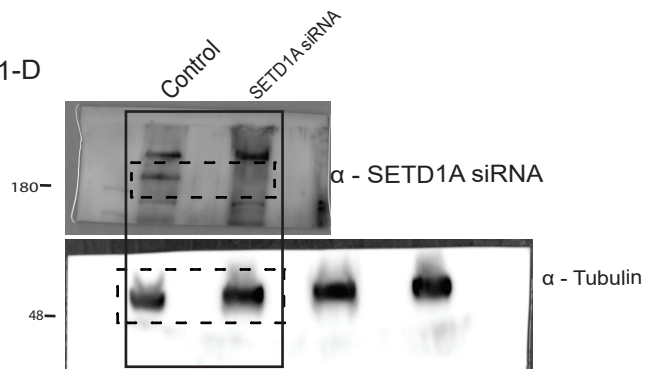

S1-E

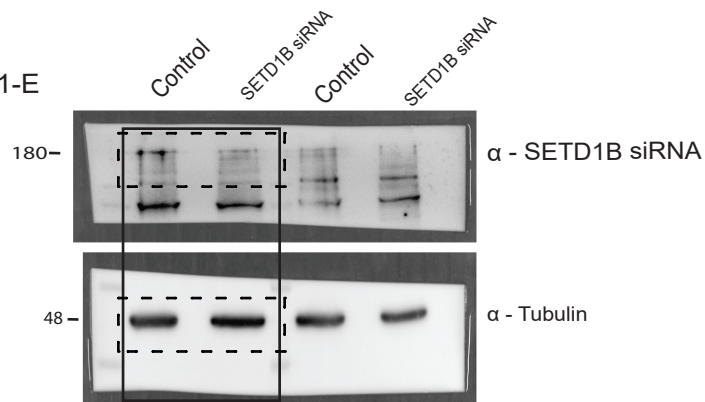

S2-G

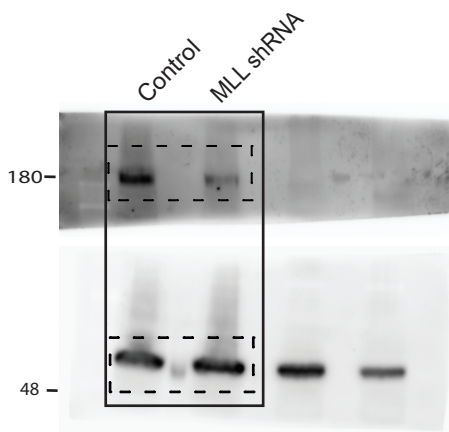

S2-I

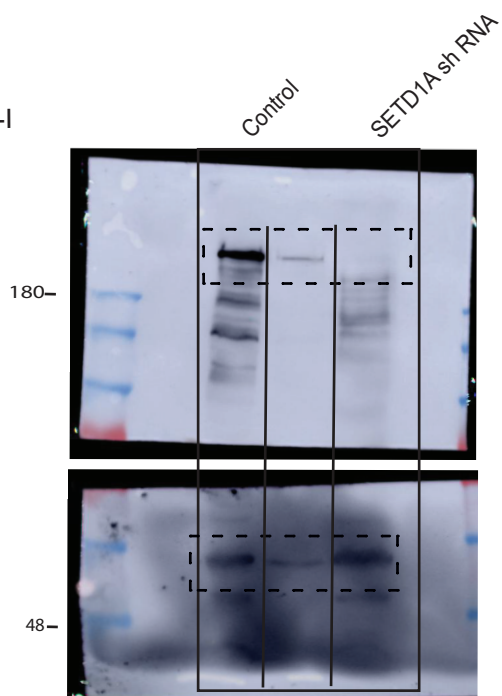

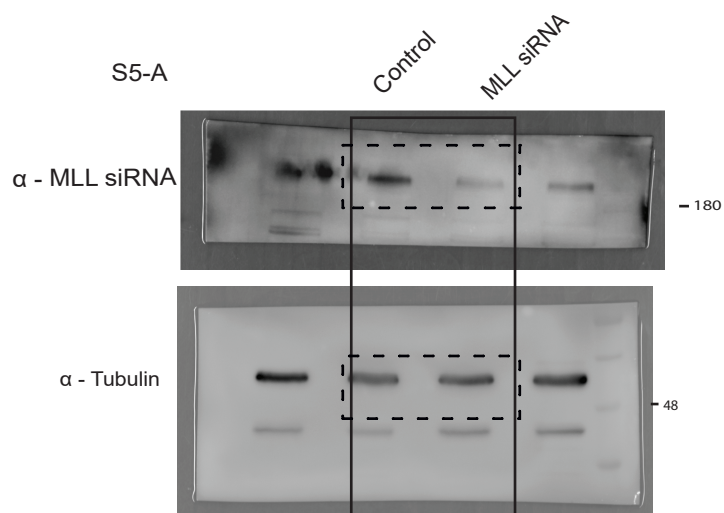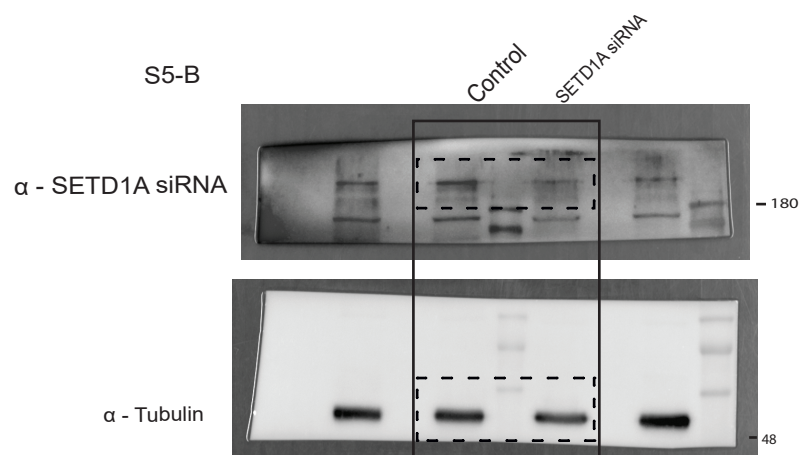

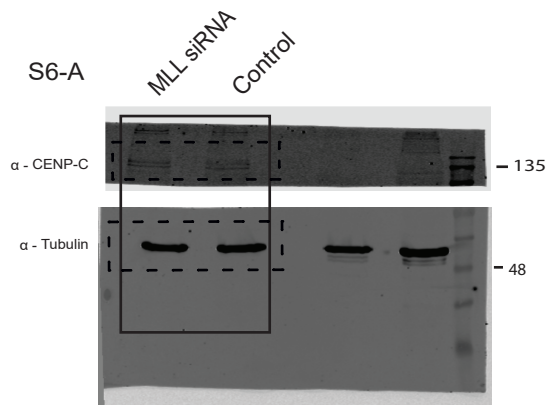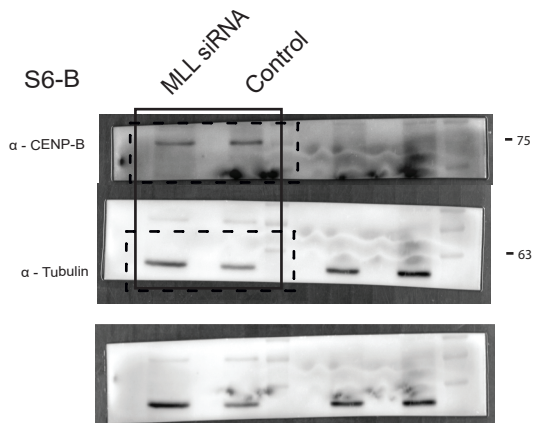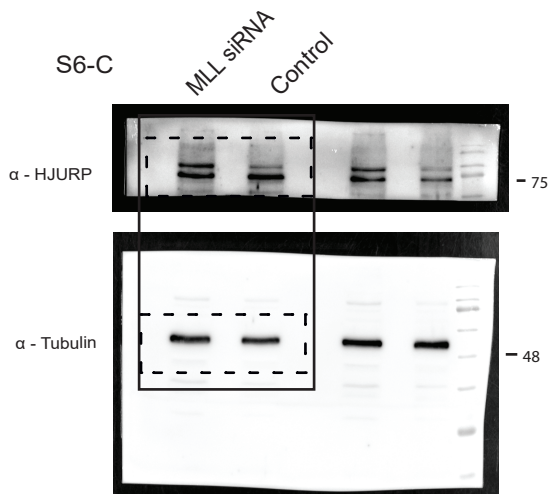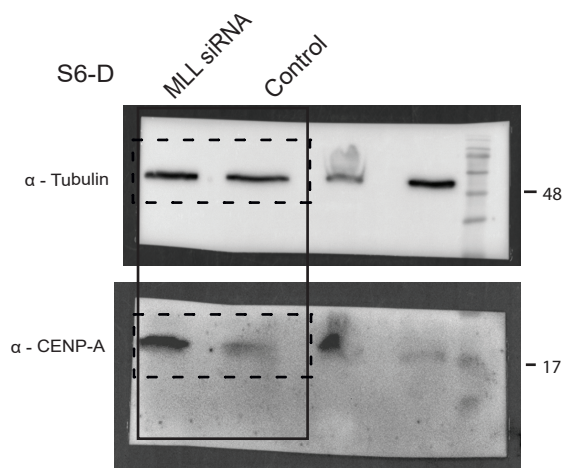

Tubulin blot is common for S6-E,G

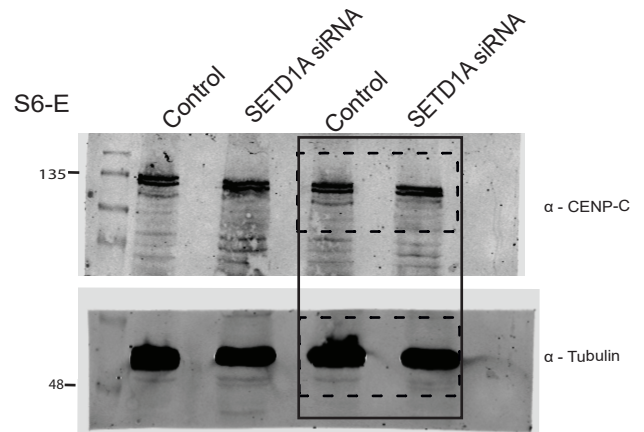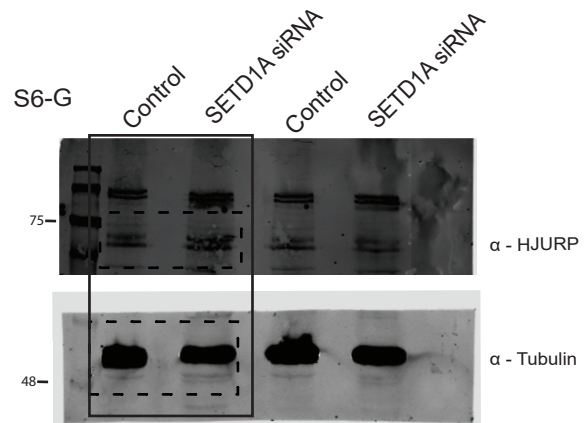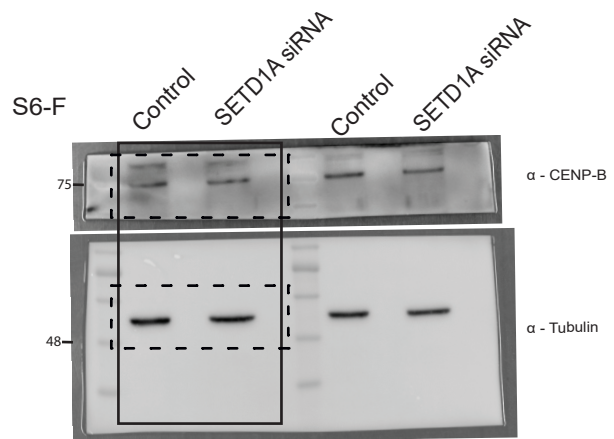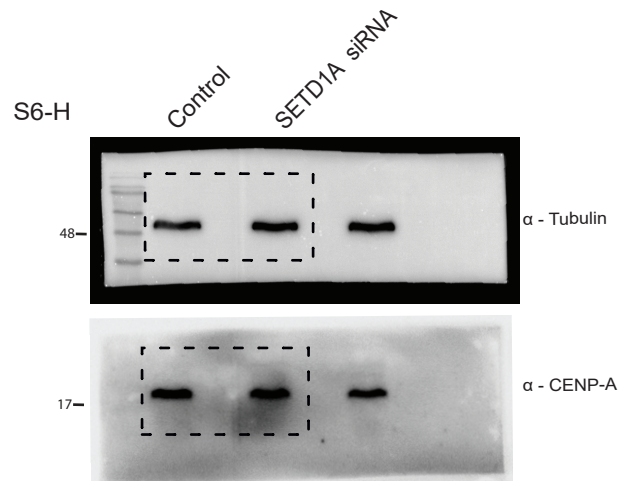

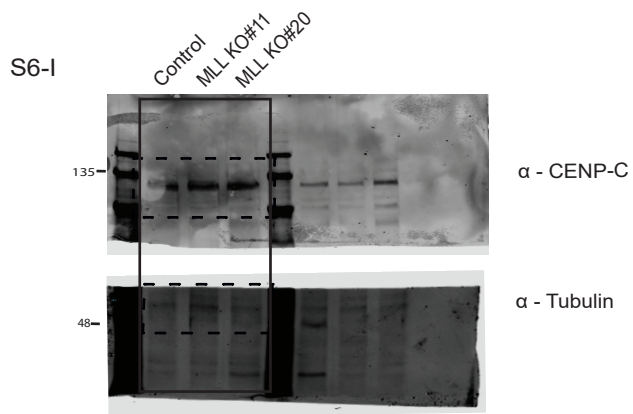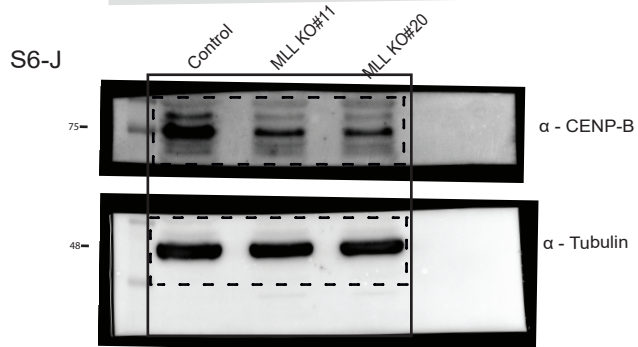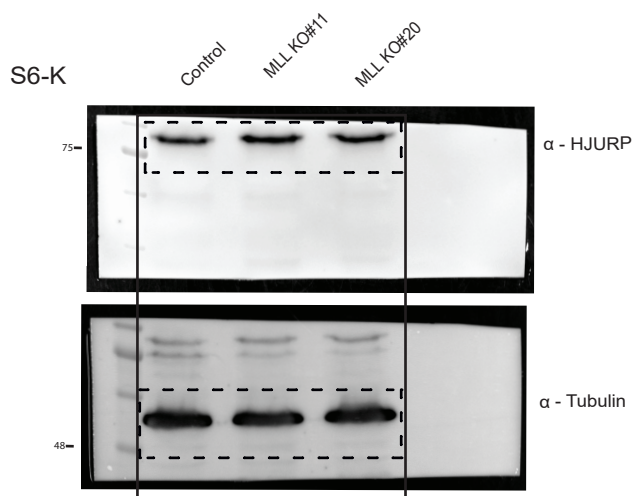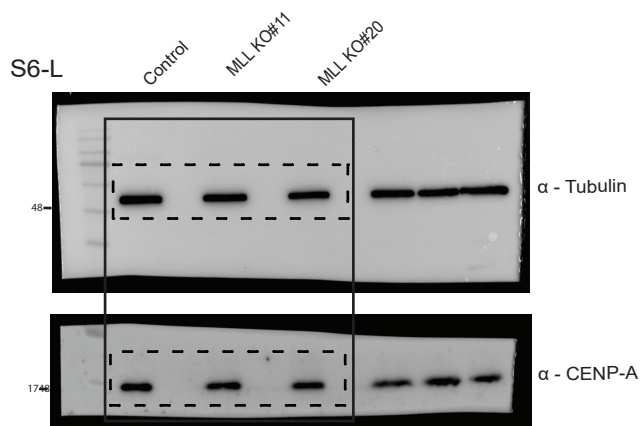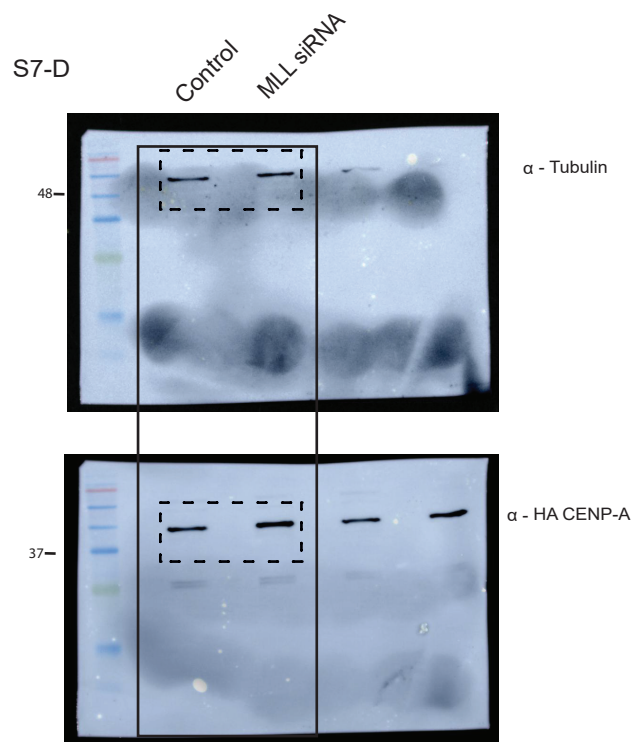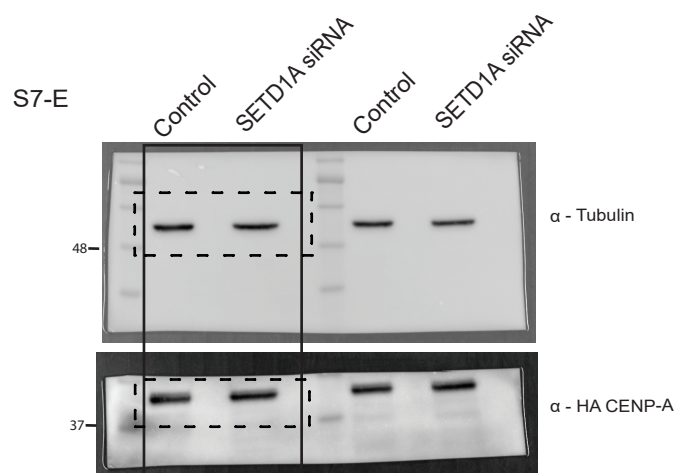

Supplement: S1 Raw Images — (PDF) [file pbio.3002161.s014.pdf]
