# Supplementary material for: MLL methyltransferases regulate H3K4 methylation to ensure CENP-A assembly at human centromeres
Source: PLoS Biol. 2023 Jun 28;21(6):e3002161. doi: 10.1371/journal.pbio.3002161 (PMC10335677; doi:10.1371/journal.pbio.3002161)
Supplement: S1 Methods — (DOCX) [file pbio.3002161.s008.docx]

**Supplemental Methods:**

**shRNA cloning and transfections:**

shRNAs constructs for MLL and SETD1A were generated by cloning oligonucleotides (sequences given below) in EcoR1 and Kpn1 linearized pLKO.1 vector (Sigma). SETD1A shRNA#2 was a gift from Suming Huang [1]. To deplete MLL and SETD1A using shRNA, HEK 293 cells were transiently transfected twice with MLL shRNA #1 and # 2 or SETD1A shRNA #1 and # 2 using PEI. Scrambled shRNA was used as Control. Samples were collected 72 hr after the first round of transfection.

**Cell Cycle Analysis**

For cell cycle analysis HEK-293 cells were harvested, washed in PBS and resuspended in 70% chilled methanol overnight. Next day, cells were washed twice in cold PBS and diluted in propidium iodide (50µg/ml), RNase (10µg/ml) and incubated for 30 min at 37 °C. Samples were analyzed with flow cytometry (BD LSRFortessa Cell analyzer) and Flowjo software.

**shRNA sequences used in this study**:

| **Name** | **Sequence** | **Source reference** |
| --- | --- | --- |
| MLL shRNA#1 | 5’-GCCTCCATCAACAGAAAGGAT | This study |
| MLL shRNA #2 | 5’- CTACCAACCCTAAACCCTG | [2] |
| SETD1A shRNA #1 | 5’-AGCAAAAGGGACCCACCCC | This study |
| SETD1A shRNA #2 | 5’GACAACAACGAATGAAATA | [1] |
| Control (Scrambled) | 5’-GCGCGATAGCGCTAATAATTT | [2] |

**siRNA sequences used in this study**:

| **Name** | **Sequence** | **Source reference** |
| --- | --- | --- |
| MLL siRNA #1 | 5’-AAGGAAAGCAUUACUGAGAAAUU | [3] |
| MLL siRNA #2 | 5’-ACGAAAGACTGAATGTAAAUU | [3] |
| SETD1A siRNA^SR^ #1 | 5´-GGAAAGAGCCAUCGGAAAUUU | [3] |
| SETD1A siRNA #2 | 5´-GCAAGAUGGUGGAGAACGUUU | [3] |
| SETD1B siRNA | 5’-GAUGAGAACCAAUGAGUUUUU | This study |
| MLL2 siRNA #2 | 5´-GGAUGAAGUUAGAGAAAAUUU | [3] |
| MLL3 siRNA #2 | 5´-GGAUAGAGCUAAGGGAUAAUU | [3] |
| Luciferase siRNA | 5´-CGUCGCGGAAUACUUCGA | [3] |

**The sgRNAs used for MLL are as follows**:

| **Name** | **Primer designation** | **Sequence** |
| --- | --- | --- |
| MLL sgRNA 1 | Fw | CACCGACATGGCGCACAGCTGTCGG |
|  | Rv | AAACCCGACAGCTGTGCGCCATGTC |
| MLL sgRNA 2 | Fw | CACCGCGAACATGGCGCACAGCTGT |
|  | Rv | AAACACAGCTGTGCGCCATGTTCGC |

**The sequence of primers used in this study for ChIP, DRIP, and transcript analysis:**

| **Gene** | **Primer designation** | **Sequence** | **Application** | **Source or reference** |
| --- | --- | --- | --- | --- |
| *α-satellite* | Fw | CATCACAAAGAAGTTTCTGAGAATGCTTC | ChIP, DRIP, and transcript analysis | [4] |
|  | Rv | TGCATTCAACTCACAGAGTTGAACCTTCC |  |  |
| *D17Z1* | Fw | CTTTGGATGGAGCAGGTTTGAGAC | ChIP, DRIP, and transcript analysis | [5] |
|  | Rv | CGTTTAGTTAGGTGCAGTTATCC |  |  |
| *D17Z1-B* | Fw | CACTGTTTGGCCTTCGTTC | ChIP, DRIP, and transcript analysis | [5] |
|  | Rv | TCCACTTGCAGATTCCACA |  |  |
| *D17Z1-C* | Fw | GCCTATGGTACTAAAGGGAAT | ChIP, DRIP, and transcript analysis | This study |
|  | Rv | ATCCTCAGAGAGGTCCAAAT |  |  |
| *HOXA9* | Fw | CTCCGCCGCTCTCATTCTCAG | ChIP | This study |
|  | Rv | GCCAGAAGGGGTGACTGTCC |  |  |
| *PAX3* | Fw | AGCCGCATCCTGAGAAGTAA | Transcript analysis | This study |
|  | Rv | CAGCTGTTCTGCTGTGAAGG |  |  |
| *PAX9* | Fw | CCGCATGACAGATTTTGCTA | ChIP | This study |
|  | Rv | GCGTTTGGTCTGAATGTGAA |  |  |
| *RAD18* | Fw | ATGCGCAGTACAAGCCCTTA | ChIP | This study |
|  | Rv | GCTCCAACACCACTCGAAAT |  |  |
| *APOL4* | Fw | GACGCCCTTTGATTGCTTGG | Transcript analysis | This study |
|  | Rv | CCAGGTTCCGGGCTACTTTT |  |  |
| *HBB* | Fw | GTGAGCTGCACTGTGACAAG | Transcript analysis | This study |
|  | Rv | TGGTGGGGTGAATTCTTTGC |  |  |
| KDM5A | Fw | CCAGGCACAAGGATGAACATTC | Transcript analysis | This study |
|  | Rv | TTTGTTCCCATTGCCAAGCC |  |  |
|  |  |  |  |  |
| SSRP1 | Fw | ATCAAGCAAGGCCAAACTCG | Transcript analysis | This study |
|  | Rv | AAGCGCTTCTCCACTTCTTC |  |  |
| ATR | Fw | TGCTGACGTGCGAAAACAAG | Transcript analysis | This study |
|  | Rv | TGGCAGCAAGATCAGGTAGTAG |  |  |
| TP53BP1 | Fw | TGCAAAGCAGCTAAGCTCAG | Transcript analysis | This study |
|  | Rv | AGAAGCTTTGTGAGGCATGG |  |  |
| MIS18A | Fw | AGGAGGACACCAACTGCATC | Transcript analysis | This study |
|  | Rv | CACGTAGCCAAGATTGAGTGAG |  |  |
| MIS18B | Fw | ATCGCTCACGTTGTGCAAC | Transcript analysis | This study |
|  | Rv | CCATGGAGGTCGTAAAAGAAGC |  |  |
| MIS18BP1 | Fw | AACTGCCAAAGTGGGAACTC | Transcript analysis | This study |
|  | Rv | TGTCCTGGAAACTTGGCAAC |  |  |
| CENPN | Fw | TGTGAGGAAAAGCGTGCAAG | Transcript analysis | This study |
|  | Rv | CCCAAACTTTCTGGTGCTGATG |  |  |
| CENPI | Fw | TGCCGTGAAGCAAAGAAACC | Transcript analysis | This study |
|  | Rv | AGAGGACGAACATTAGCTGGAC |  |  |
| CENPO | Fw | AGCACTTCCTGTTCAGTCTCTG | Transcript analysis | This study |
|  | Rv | GCTGCAAAGTCACTCTGAAGC |  |  |
| CENPL | Fw | GCTGGTTCTGCTGTGTATTTGG | Transcript analysis | This study |
|  | Rv | AAGGAATAAGGGCAGACAGGTG |  |  |
| CENPT | Fw | AGGTCAATGCCTTTGCTCTG | Transcript analysis | This study |
|  | Rv | TGCCTCCATTTCACTCACAC |  |  |
| AURKB | Fw | AGGTGATGGAGAATAGCAGTGG | Transcript analysis | This study |
|  | Rv | AAACTTGCCTTTGCCCAGAG |  |  |
| ZFAT | Fw | AGCTGGCAGAAAACATCGTG | Transcript analysis | This study |
|  | Rv | AGAACTTCCGACAGCACTTG |  |  |
| PDS5A | Fw | TCTGCCAATTCAACCCTTCG | Transcript analysis | This study |
|  | Rv | AACAAGGTTCCTGAGCAAGC |  |  |
| CD180 | Fw | GGCAGCTTGGAGGTTCTGAT | Transcript analysis | This study |
|  | Rv | ATGTCAGGCTGTTGTGGCTT |  |  |
| PRIM1 | Fw | TCCACGGCTGGATATCAATGTC | Transcript analysis | This study |
|  | Rv | CCAATTCACGGCAGATGAAGC |  |  |
| RPA3 | Fw | GAGGCTGGAAAAGATTCATCCC | Transcript analysis | This study |
|  | Rv | TTGGCGGTTACTCTTCCAAC |  |  |
| MESI1 | Fw | GTCACACAGTGGGGACAACA | Transcript analysis | This study |
|  | Rv | CAGCCACGCCCTCATGATAT |  |  |
| MYL4 | Fw | CAGCACATTTCCCGCAACAA | Transcript analysis | This study |
|  | Rv | GCATCCTCTTGCCCAGCTAA |  |  |
| *CCTT5* | Fw | CCCTTCCCTCCCATTCCA | Transcript analysis | [6] |
|  | Rv | AGACATCTGGTTGCCCTCC |  |  |
| *RPL13A*  *(Intron 7)* | Fw | AGGTGCCTTGCTCACAGAGT | DRIP | [7] |
|  | Rv | GGTTGCATTGCCCTCATTAC |  |  |
| *RPL13A*  *(Exon 8)* | Fw | GAGCAAGGAAAGGGTCTTAG | DRIP | This study |
|  | Rv | CTTCTAGAAATACCCTGTGTAC |  |  |
| *MLL* | Fw | GGAGCACACATTCCAGACCA | Transcript analysis | [3] |
|  | Rv | TTTGGGTCACCTGAACTTCC |  |  |
| *MLL2* | Fw | AGCCGTGTGAGGATGAAAAC | Transcript analysis | [3] |
|  | Rv | ACCTGGGGAGGACCATCTT |  |  |
| *MLL3* | Fw | CAGCACCACGAAAACAAAGA | Transcript analysis | This study |
|  | Rv | ACTCCACACAACGGTGATGA |  |  |
| *SETD1A* | Fw | CGAATACGTGGGTCAGAACA | Transcript analysis | [3] |
|  | Rv | TGCAGCAGTGGTTGATGAAT |  |  |
| *SETD1B* | Fw | TGGACACCAAAGGGGAAACC | Transcript analysis | This study |
|  | Rv | CAGACAGGCCTCCATCCTTG |  |  |
| *GAPDH* | Fw | CGAGATCCCTCCAAAATCAA | Transcript analysis | This study |
|  | Rv | TTCACACCCATGACGAACAT |  |  |
| *U2_C_* | Fw | TTTGCTCCCACTGCCGTC | ChIP and DRIP | [8] |
|  | Rv | CTGAGTCTTTCGGTGCCC |  |  |
| *CD4* | Fw | TCTGCAGAAGGAACAAAGCA | ChIP | This study |
|  | Rv | GGAAGGAAGCCGAGTCTGA |  |  |
| *EGR1* | Fw | GAACGTTCAGCCTCGTTCTC | DRIP | [7] |
|  | Rv | GGAAGGTGGAAGGAAACACA |  |  |
| *SNRPN* | Fw | TGCCAGGAAGCCAAATGAGT | DRIP | [9] |
|  | Rv | TCCCTCTTGGCAACATCCA |  |  |

**Reference:**

1. Deng, C. *et al.* USF1 and hSET1A Mediated Epigenetic Modifications Regulate Lineage Differentiation and HoxB4 Transcription. *PLoS Genet.* **9**, (2013).
2. Liu, H., Cheng, E. H. Y. & Hsieh, J. J. D. Bimodal degradation of MLL by SCFSkp2 and APCCdc20 assures cell cycle execution: A critical regulatory circuit lost in leukemogenic MLL fusions. *Genes Dev.* **21**, 2385–2398 (2007).
3. Ali, A., Veeranki, S. N. & Tyagi, S. A SET-domain-independent role of WRAD complex in cell-cycle regulatory function of mixed lineage leukemia. Nucleic Acids Res. 42, 7611–7624 (2014).
4. Quénet, D. & Dalal, Y. A long non-coding RNA is required for targeting centromeric protein A to the human centromere. Elife 3, e03254 (2014).
5. McNulty, S. M., Sullivan, L. L. & Sullivan, B. A. Human Centromeres Produce Chromosome-Specific and Array-Specific Alpha Satellite Transcripts that Are Complexed with CENP-A and CENP-C. Dev. Cell 42, 226-240.e6 (2017).
6. Zhang, C. *et al.* LncRNA CCTT-mediated RNA-DNA and RNA-protein interactions facilitate the recruitment of CENP-C to centromeric DNA during kinetochore assembly. *Mol. Cell* **82**, 4018-4032.e9 (2022).
7. Sanz, L. A. & Chédin, F. High-resolution, strand-specific R-loop mapping via S9.6-based DNA–RNA immunoprecipitation and high-throughput sequencing. Nat. Protoc. 14, 1734–1755 (2019)
8. Zargar, Z. U., Kimidi, M. R. & Tyagi, S. Dynamic site-specific recruitment of RBP2 by pocket protein p130 modulates H3K4 methylation on E2F-responsive promoters. Nucleic Acids Res. 46, 174–188 (2018).
9. SridharaS. C. et al. Transcription Dynamics Prevent RNA-Mediated Genomic Instability through SRPK2-Dependent DDX23 Phosphorylation. Cell Rep. 18, 334–343 (2017).
