# Supplementary material for: MLL methyltransferases regulate H3K4 methylation to ensure CENP-A assembly at human centromeres
Source: PLoS Biol. 2023 Jun 28;21(6):e3002161. doi: 10.1371/journal.pbio.3002161 (PMC10335677; doi:10.1371/journal.pbio.3002161)
Supplement: Attachment — Submitted filename: Response to Reviewers.pdf [file pbio.3002161.s017.pdf]

Reviewer (them/ their)

Overall description of modifications made to the manuscript in response to the reviewers' comments

We thank all the Reviewers for their insightful comments and suggestions. Overall we noted that the Reviewers were happy with the quality of our data and controls there in but wanted additional experiments to affirm conclusions. Based on their suggestions, we have added more experiments to the manuscript. This has led to addition of more figures (7 main and 7 supplementary figure with three tables. One figure is added here also). To aid the reviewers in evaluating the revision, we have first listed the key changes made to the manuscript and then describe point by point how we have addressed each of the reviewers' comments.

Based on Reviewers suggestions, the key experiments we have performed are as follows:

1. Double KD of MLL and SETD1A to assess for synergistic/contrasting affects in recruitment of CENP-A and R-loops.
2. Kinetochore fibre co-staining between H3K4me2 and CENP-A in presence and absence of MLL/SETD1A to support our present experiments.
3. Additional experiments on R-loops and Pol II recruitment to provide better clarity on the disparity observed between MLL and SETD1A loss.
4. RNA-seq experiments to assess the transcriptional changes upon MLL or SETD1A KD.

Pleasingly, majority of these experiments have confirmed our hypothesis further.

Reviewer #1 (Evidence, reproducibility and clarity (Required)):

Summary
The manuscript "KMT2 family members regulate H3K4 methylation to ensure kinetochore activity at human centromeres" by Malik and colleagues describes a study of MLL and SETD1A role on centromeric transcription. Previous work showed that centromeric chromatin is marked by a distinct PTM signature, including H3K4me2. Therefore, every KMT2 family member is knocked-down and centromeric transcript levels are determined. From here, the authors focus on just two of the KMT2 family members: namely MLL and SETD1A. In addition to qRT-PCR, the authors do ChIP-PCR to confirm that MLL and SETD1B also associate with centromeric DNA, as well as a loss of centromeric H3K4me2. Subsequently, centromeric RNAP2 and R-loops (DNA-RNA hybrids) were analysed. Finally, a series of IF experiments are performed using FL or truncated version of MLL or SETD1A to rescue KD phenotypes. By IF levels of kinetochore proteins and CENP-A nucleosomes were analysed. Altogether, the KTM2 members MLL and SETD1A impact centromere chromatin landscape both at the level of PTMs and kinetochore protein recruitment and retention.

Major comments:

I think the experiments within the manuscript are generally of good quality and well controlled. However, I find that the presentation and description of the results were at times confusing. In fact, to get a better grip on the data, I made summary tables to get a better sense of what the

data was telling. In my opinion, this manuscript contains findings that are of interest to the field, even when the results might now be clear cut. Personally, I would encourage the authors to consider a follow up where combinations of KMT2 family members are KD to hopefully get a cleaner picture of how nucleosome methylation is regulated at the centromere and how this regulates centromere identity.

We thank the Reviewer for their comments. With this review process, and suggestions from all four Reviewers, we hope that the manuscript is adequately presented with clear description of the results.
We have taken Reviewer's suggestion and performed experiments where MLL and SETD1A was KD simultaneously and the cells were assessed for i) CENP-A/HJURP levels (Figure 6G-H, S7A), ii) R-loop formation (Figure 4A). We find that MLL and SETD1A are synergistic in their actions on CENP-A recruitment but R-loop resolution seems entirely dependent on MLL.

+ The title does not reflect the data presented in the manuscript, as kinetochore activity is not tested.

We received this criticism from other Reviewers also. We have now changed the title to aptly reflect the data presented in the manuscript. The new title is as follows " MLL family members regulate H3K4 methylation to ensure CENP-A assembly at human centromeres."

+ Several cell lines (HEK293, IMR90, and U-2OS) were used to study MLL and SETD1A, but the rational why these cell lines were used and why one cell line was used in one study, but not another is not clearly justified.

When working with cell lines, in the field, it is generally recommended to try our assays in different cells in order to remove a cell-line specific bias and gauge the function of protein in a general Vs cell-line specific phenomenon. In the same spirit, we have used multiple cell lines. Further, U-2OS cells are generally preferred for IF whereas, HEK-293 are preferred for ChIP/DRIP. IMR90-tert are problematic for both, but as these are non-cancer cells, we used them in our ChIP assays to determine binding of MLL in 'normal cells'.

Further, even though we tried to generate iKOs in multiple cells, we only achieved success in HEK-293 cells.

Our mutant cell lines were previously made and validated in U-2OS. Hence, we used those for transcript and IF studies.

We have added these statements to the manuscript.

+ Did KD of all the KMT2 family members also result in noticeable reduction in protein levels?

We had only checked the protein levels of MLL and SETD1A by IF and WB after siRNA KD. Now we have incorporated blots showing protein levels of MLL2 and SETD1B, which show reduction upon siRNA treatment (Figure S1D-E). MLL3 is a large protein (5000 aa) and does not get processed. In

the past, we have not been successful in obtaining MLL3 band on WB. Therefore, we did not attempt to check its protein levels here.

+ The authors frankly state that the antibody S9.6 is not the ideal method to detect DNA:RNA hybrids, so they do DRIP experiments as well. Yet, DRIP still relies on S9.6. It might therefore be worthwhile to consider using catalytically dead RNaseH fused with GFP, as described in Crossley et al JCB 2021.

While we appreciate the relevance of this opinion, we hope that the Reviewer will agree that this experiment will require considerable time and effort and yet, not add extra value to our current findings. Moreover, previous reports suggest a disparate bias towards these approaches i.e., DRIP and R-ChIP (ChIP with dead RNaseH) in mapping genomic R-loops (Chedin et al 2021, PMID: 33411340). This is the reason we carried out the widely accepted **classical DRIP** experiment by incorporating RNase H as a control, to validate our IF results.

We will surely consider this suggestion in our follow up studies when we attempt to understand how MLL resolves R-loops formation.

+ The data is confusing based on previous reports. H3K4me2 is presented as being necessary for centromeric transcription and subsequent maintaining centromere identity. Yet, KD of MLL does not impact RNAP2 levels, increases activated RNAP2, but H3K4me2 levels are significantly down. In contrast, SETD1A KD has a different picture.

The Reviewer has nicely summed up the conundrum that we wish to highlight. Based on previous and our report, H3K4me2 is indeed necessary for centromeric transcription and subsequent maintaining centromere identity. What we wish to highlight is that two HMTs from the same family, which deposit the H3K4me2 mark, may differ in other aspects to influence the stability of the centromere differently. This is manifested in the unexpected paused RNA Pol II levels and accumulation of R-loops upon KD of MLL.

Now we have added new data, which shows that (i) R-loops are MLL-specific and even in double KD of MLL and SETD1A, they exhibits same level of accumulation as MLL alone (Figure 4A); (ii) KD of SETD1A has opposite effects on RNA Pol II levels than MLL KD (Figure 4F-G). Both total RNA Pol II and RNA Pol II^{S2P} levels are reduced on the centromeres upon SETD1A, confirming our initial hypothesis.

Our findings underscore the fact that both MLL and SETD1A may interact differently with the RNA Pol II during (centromeric) transcription and their loss has distinct effect on the recruitment/elongation of RNA Pol II. As most pioneering studies on H3K4 HMTs relationship with RNA Pol II were done in yeast (Set1A), how different KMT2 members may influence this relationship in humans is relatively under studied.

+ The data presented in Figures 5 and 6 were confusing, which is in part due to unclarity of why when what cell line was used. U-2OS is shown as the base-line, as if the addition of MLL-FL, MLLdTAD, MLLdSET are rescue constructs. Why in addition also knock-down MLL. Would this also knock-down the rescue constructs, or only the native proteins? It would be very helpful if these figures are made much easier to interpret.

We thank the Reviewer for their comment and apologize for leaving out this crucial information. U-2OS is indeed base-line and MLL-FL, MLLΔTAD, MLLΔSET are rescue constructs. The KD of endogenous wild type transcript is necessary to gauge the effect of mutant proteins alone.
We have now explained in the text how our siRNA is directed against 3' UTR of precursor mRNA for MLL (MLL siRNA #2), which only affects endogenous mRNA. Recombinant cDNA constructs do not have 3'UTR, hence remain unaffected by it.

For SETD1A, we did not have siRNA against 5' or 3'UTR of precursor mRNA. Therefore, we made recombinant SETD1A constructs, resistant to our siRNA by introducing silent mutations in the region of SETD1A, which will bind the siRNA (Figure S1I, SETD1A siRNA#1) by PCR mutagenesis. As a result, transcripts from all the cDNA constructs of SETD1A used here, will not be affected by SETD1A siRNA#1.

+ Visual inspection of western blots do not appear to be consistent. MLL KD as shown in Fig S2C is less efficient compared to Fig3.

We would like to draw the attention of the Reviewer towards the fact that Fig S2C shows transient **knock-down** of MLL using shRNA whereas Fig3A shows levels of MLL in our inducible **knock-out** cells. shRNA treatment-mediated knock down is less efficient and that is also reflected in our blots.

Similarly, HA-CENP-A levels are up in Fig S5D, G, H. This appears to be contradictory to the premise of this manuscript that H3Kme2 is essential for guaranteeing centromeric transcription, which in turn is essential for new CENP-A loading. Therefore, the prediction would be that KD of KMT2s would result in loss of H3K4me2 (Fig3C), loss of centromeric transcription (Fig1A) and subsequent loss of new CENP-A loading (Fig 6K,L) as well as total CENP-A (replication-loss is not replenished). Here the authors show by western blot that MLL KD results in increased CENP-A levels (Fig S5D,G,H). As the authors looked at WCE, it is possible that non-chromatinized CENP-A is being detected. It might be worth repeating these western blots for chromatin bound CENP-A only to get a cleaner picture. Additionally, all western blots should be quantified.

Our reason for looking at WCE for levels of CENP-A (and other proteins) is to check if protein levels are affected due to transcriptional role of MLL (i.e. if any of these proteins are transcriptional targets of MLL; please also see comment 3 by Reviewer #3 and our response). Therefore, the idea is to check for non-chromatinized levels of CENP-A. Chromatin bound CENP-A levels are quantified in the IF images, which show a decrease upon MLL and SETD1A KD (Figure 6). Additionally, we have added new data (in Figure 7G, S7F-G), where we have performed IF for H3K4me2 and CENP-A on stretched chromatin fibres in presence and absence of MLL or SETD1A. These experiments show that chromatin bound CENP-A levels are decreased upon KD of our HMTs.

In Figure old figure S5 G,H (new figure S7D-E), the CENP-A is recombinantly expressed from CENP-A-SNAP-3xHA construct with a CMV promoter. We have now added quantification from two independent experiments, which shows that HA-CENP-A levels remain unchanged upon MLL KD (Figure S7D). However, they do show slight but significant increase upon SETD1A KD (Figure S7E). Ideally, this exogenous expression system should not be affected by MLL/SETD1A KD. While we did not expect to see reduction in CENP-A levels in these cells, we did not expect an increase also and at the moment unable to explain these results. However, despite this increase in recombinant

CENP-A WCE levels, we observe a decrease in our IF quantification of chromatin HA-CENP-A (total CENP-A) upon SETD1A KD (new Figure S7C).

Quantification is shown for all WBs now. Crucial experiments have been quantified using data from two independent experiments.

Reviewer #1 (Significance (Required)):

Nature and Significance of the advance:

This work adds to the study of centromere chromatin, which is inherently difficult to study because of its repetitive nature. Most work done thus far on distinct PTMs in relationship to centromere function has been done on HACs. This study ventures into the more native centromere structure of established centromeres of cycling cell lines. To strengthen the manuscript, the authors could do the experiments suggested above, but above all KD both MLL and SETD1A simultaneously to see if there is synergistic interplace between the various KTM family members.

Reviewer #2 (Evidence, reproducibility and clarity (Required)):

The histone mark H3K4me2 is enriched at centromeres and is essential for centromeric transcription and proper chromosome segregation. While the importance of this mark is relatively understood, the enzymes depositing it at the centromeres are not well characterised. This paper by Malik et al., uses RNA interference to show that depletion of the KMT2 methyltransferase family members leads to reduced centromeric transcription. They then use chromatin immunoprecipitation and immunofluorescence to show that these enzymes are present at centromeric sites. By using inducible knockout (iKO) cell lines, the authors further investigate the changes in the centromeric epigenetic landscape caused by the absence of MLL and SETD1A. They observe a decrease in H3K4me2 and H3K9ac marks, and a corresponding increase in H3K36me2 and H3K9me3. In addition, an accumulation of R-loops and serine-2 phosphorylated RNA polymerase II is observed at centromeres in MLL iKO cell lines. The authors then investigate the effect on known centromeric proteins and find that upon MLL and SETD1A depletion, the levels of CENP-C, CENP-B, and HJURP are reduced, together with reduced loading of nascent CENP-A.

Major points:

1. My major concerns with this study are the potential secondary effects when you tamper with transcription of prolonged periods of time in the cells. As the authors state themselves "MLL1 (and SetDB1?) is essential for cell viability and growth" - keeping that in mind the experimental setups for siRNA experiments extend from 48-72h (couldn't find the description for CRISPR in the methods section). In any case, a lot can go wrong over the course of 3 days without proper transcription, so the authors should show evidence that cells are proliferating normally and moving fine through the cell cycle, similar to wildtype. This is also important as for instance loading of CENP-A itself requires moving from mitosis into G1. In general, faster acting ways to

deplete proteins, such as degron-based approaches would be more suitable as an experimental setup.

We thank the Reviewer for their comment. Cas-9 induction is now listed under 'Generation of Stable Cell Lines' and goes on for 7 days. We have included representative FACS profiles from MLL iKO cells to show that these cells are proliferating (Figure S3A). The profiles show minor differences between Control and Test, which are not significant statistically in three independent experiments.

Further, all the images shown for CENP-A loading are of cells released in early G1 stage (Figure 6 A-B,G, 7B).

We agree with the Reviewer that the time required to efficiently degrade MLL or SETD1A protein using siRNA method is enough to cause problems for the cell. In fact, we have ourselves reported that about 50% cells undergo stable arrest upon MLL siRNA (Ali et al., 2014, PMID: 24880690) after 72hrs and cells which enter mitosis spend long time in pro-metaphase or metaphase (Ali et al., 2017, PMID: 28633016). Taking all these factors into account, we have released cells for 60 mins (mitotic cells) or 90 mins (early G1) after nocodazole arrest and fixed them thereafter. This was to ensure that we catch only proliferating cells.

We agree with the Reviewer that the degron-based approach will indeed simplify our experimental set-up. We have been trying to obtain knock-ins for MLL-degrons but so far we have not achieved success. It is our general experience that genome editing experiments involving *MLL/SETD1A* (compared to other genes used in our lab) are not easy to accomplish. While it may become a part of our future experimental set-up, at this time, looking at our past failed attempts, we cannot plan to include such experiments in this manuscript. We would like to draw the attention of the Reviewer to the experiments performed on HAC (Bergmann et al., 2011, PMID: 21157429). Even when LSD1 expression results in rapid loss of H3K4me2, 50% decrease was observed in CENP-A after 3 days and segregation defects were observed 7 days later, indicating that loss of CENP-A can be tolerated over several divisions. This indicates that even by using the degron approach if we are able to achieve H3K4me2 depletion rapidly, we may still have to wait 3 days (same time as used here for siRNA treatment) to observe a reduction in centromere proteins.

In order to address concerns about affects on transcription upon MLL and SETD1A, and to address points raised by Reviewer#3, we have performed RNA-seq experiments upon MLL and SETD1A depletion in U-2OS cells and included these analyses in Figure S5. They show that in U-2OS cells, MLL or SETD1A siRNA mediated KD does not causes major changes in the cell cycle or centromere gene expression.

2. While the IF images for centromere localisation of MLL1 and SetD1A in mitotic cells are truly remarkable (Figure 2 and 5, etc), it is important to show an interphase staining for both proteins as well. First of all it would be interesting to see if the centromere staining persists in interphase, but more importantly, a genome wide staining would be expected and provide an important validation of the antibodies.

We thank the Reviewer for their appreciation. Now we have included interphase (Figure S2A) as well as early G1 (Figure 2B) staining. It shows that MLL and SETD1A are present on the centromere in interphase and more crucially, in early G1 phase. We would also like to draw the Reviewer's attention towards the fixation used here (1% paraformaldehyde or methanol), to

enhance the centromere staining Vs the whole nuclear staining. The validation of antibodies is provided in Figure S2C-D, where upon siRNA treatment, the staining of MLL/SETD1A is reduced on the centromere in IF, and Figure S2G-J, where shRNA treatment reduces MLL/SETD1A signal for WB and ChIP.

3. H3K9me3 changes do not always correlate with H3K4me2 decrease (not all loci increase when H3K4me2 decreases). Could you please comment on this? Also, there is a massive difference between iKO#11 and #20 when looking at H3K9me3 levels. In figure S3D, it is very difficult to interpret the differences due to the split bar.

We agree with the Reviewer on this. Even though the final outcome was similar, there seems to be a wide variation in the reading of iKO#11 and iKO#20, mostly due to noticeable variation in qPCRs. We have performed additional ChIP experiments and shown data from three closely related experiments. All loci now show correlative increase in H3K9me3, and the readings in iKO#11 and #20 are closer to each other.

4. I'm confused about the conclusions shown in figure 4A. Here, siRNA of SetD1A is compared to siRNA of SetD1A + Triptolide and show no significant difference. This is used to argue that the changes in R-loop formation is mainly transcription based. However, if one compares the 2 control lanes, 1 and 4, is noticeable that they are almost identical and predictably not significantly different. Does this mean that addition of triptolide and consequently inhibition of transcription has no effect on R-loop formation in their experiment.? The authors should explain this. In addition, it also looks like there a reproducible difference between SetD1A siRNA alone and with triptolide. This should be discussed, too.

We thank the Reviewer for noticing this discrepancy. According to our observation/hypothesis, the Control and Control + Triptolide should be different. We have checked our other data sets and there is a decrease in Control + Triptolide sample with respect to Control. However, for some reason, the data sets displayed here do not reflect that. Based on other Reviewer's comments, we investigated the status of R-loops in double KD for MLL and SETD1A. Pleasingly, the new data set appropriately reflects our hypothesis. All relevant data sets are compared to each other. SETD1A siRNA and SETD1A siRNA + Triptolide shows no significant change statistically as indicated in the previous and new Figure 4A.

5. In figure S4B, samples are expressed as percentage of input. They are above 100%, even 500% in some cases. This would mean that more DNA than the input DNA is pulled down in the assay, which is not possible. It is likely that input sample Ct values have not been adjusted for the dilution factor in the assay. I would suggest checking these calculations again. If the other samples have been treated in the same way before calculating fold enrichment, they should be checked too.

We thank the Reviewer for pointing out this lapse. As suggested, we have corrected the values and replotted the graph S4B which now falls in the range as expected (we used similar correction formula to calculate '% Input' as in Sanz and Chedin, 2020, PMID: 31053798). The data in Figures 4B and 4C will remain the same, as they are relative enrichment values compared with control cells.

6. Fig 4D Hox9 is not statistically significant, while 4E 17Z1 is significant. Is this correct? As

mentioned before, it is not clear what the significance has been calculated against, whether against the control or the rest of the loci.

It is correct. The significance has been calculated against mean of Control Vs mean of Test. However, when we do it against rest of loci, the significance still remains the same as with above method.

7. In figure 5C, MLL-FL control sample shows reduced amounts of CENP-C compared to both the MLL siRNA treated cells and the rest of the samples. In addition, all control samples expressing MLL seem to have a general reduction in CENP-C signals compared to U2OS cells. This seems to indicate that expression of MLL might rather decrease the amount of centromeric CENP-C. Similarly, in figure 5D, expression of SETD1A leads to an increase of CENP-C signal, even in the case of SETD1AΔSET. These results indicate that SETD1A itself and not its methyltransferase activity contribute to CENP-C recruitment, which is partially in contradiction with the results shown before. Could you please comment on this?

As we have stated in the manuscript, we had problems staining for centromeric proteins in our mutant cell lines, particularly MLL recombinant constructs. As a result, some of these experiments were not done together, introducing variation in the staining (Jonkman et al., 2020, PMID: 32235926) and quantification. As this issue was highlighted by two Reviewers, we repeated imaging of some data sets, with parent U-2OS as reference (as they ideally should have been done). Further, our past multiple attempts have made us proficient in staining mutant cell lines. We observe that most mutant cell line Controls are now in the similar range as parent U-2OS. Therefore, the variations were introduced by technical error. More importantly, upon siRNA treatment of these cell lines, our previous observations still stand.

8. Figure 6C, MLL-FL cells show reduced HJURP signal in the control compared to U2OS cells. Has the protein expression of these constructs been assessed (same applies to the mutants and SETD1A)? There is a chance that these unexpected differences might come from expression differences.

The HJURP signal in control and MLL-FL cells have been corrected as described above.

As to Reviewer's suggestion of assessing the expression levels of the recombinant proteins, in our past experience, this has proved very difficult as we had purposefully selected cell lines which showed expression similar to cellular levels (Ali et al., 2014, PMID: 24880690). Therefore, we have used FLAG-epitope IF to indicate that the recombinant proteins are expressing in our cell lines (Ali et al., 2014, PMID: 24880690) and this was confirmed recently (Chinchole et al., 2022, PMID: 36111497)

9. In the discussion, the increase in S2ph in MLL knockout cells is used to suggest an aberrantly paused RNA polymerase which would lead to reduced centromeric transcription. While this is possible, MLL mutants are known to accumulate RNA pol II at transcription termination sites, and since the authors show that markers associated with active transcription (S2P and H3K36me2) are accumulated in their MLL iKO lines, additional evidence is required to support a paused polymerase. Since RNA polymerase S5ph is generally considered a marker for paused polymerases, especially close to promoters, where H3K4me2 is mostly deposited, I would suggest testing enrichment of S5P and some known RNA polymerase pausing factors (e.g. NELF, DSIF) at the centromeres either by IF or ChIP in the MLL knockdown lines.

We thank the Reviewer for this suggestion. As the status of S5P on the centromeres is not clear (Chan et al., 2012, PMID: 22308327), we planned to use NELF-E to validate paused RNA polymerase at the centromeres by ChIP in the MLL iKO cell lines. However, we ran into some logistical and technical glitches. First it took us considerable time to procure the NELF-E antibody (over 3 months). When it did finally arrive, we are unable to see the enrichment of NELF over IgG in our ChIP experiments (See Reviewer Figure 1A). We have raised a complaint with the vendor and ordered another antibody which is yet to reach us. So we have decided to submit the revision without performing this experiment.

We will certainly use this suggestion in our follow up studies.

Minor points:

1. The paper repeatedly ignores previously published work on centromere transcription in model organism like S. pombe, which spear headed research in centromere transcription (Ekwall and Allshire labs) and in Drosophila on CENP-A stability (Heun lab). These should be cited as centromere transcription is conserved in evolution and does not just exist in human cells.

We apologize for this omission. We have now included these citations in the manuscript.

2. Figure S2G. Is there truly enrichment compared to IgG, or is it different between centromeric loci and control genes? It is not clear whether significance is indicated compared to IgG or between centromeric loci or control samples. This applies to most plots. I would mention it either in the legend or indicate in the plot if possible.

There is enrichment compared to IgG. The significance is calculated against mean of test genes Vs mean of IgG. As it is uniformly applied to all plots, we have now mentioned it in the Methods section.

3. In the plots containing a break in the Y axis, the unit immediately after the break should be indicated. While the reason for introducing the break is clear given the differences between samples, the lack of that unit hampers correct comparison between samples on the two sides of the break.

We have corrected the plots and shown units on both sides now.

4. In all plots with y-axis written as "Fold over Control", I would suggest indicating what is assessed in the axis label or at least in the title (like in figure 3). Having to look for it in the text or in the legend make the figures a bit confusing.

We have corrected as advised.

5. Two sets of different siRNAs and shRNAs have been used for MLL1 and SETD1A knockdown. Is there a reason for this change? If so, I would suggest mentioning this in the text.

We have done most of our studies with siRNAs. For ChIP, however, we needed a greater number of cells. Hence, we used shRNA-transfected cells to obtain KD of our proteins.

We have added this in the text.

6. I would suggest quantifying the knockdown efficiencies and indicate these underneath the western blot panels.

We have made this correction.

7. It would be better to add the siRNAs sequences in the methods, instead of just referencing them.

We have added the siRNA sequences in the Supplementary Information.

8. Immunofluorescence is generally shortened as IF. I would suggest keeping this consistent with known literature.

We have made this correction.

9. In Figure S2A, scale bars are different between MLL1 and SETD1A. Since MLL1 siRNA cells seem to have a chromosome de-condensation phenotype or larger cells, it might help to have all images with the same magnification to allow proper comparison.

The cells are at the same magnification but the scale bar was incorrectly drawn. We thank the Reviewer for noticing this anomaly. It is now corrected.

10. Why do non transformed cells (IMR-90 tert) have much less enrichment of MLL, SETD1A, H3K4me2? Is the cell line problematic in ChIP assays, or do you think there are physiological differences?

Probably both factors contribute to the less binding observed for our proteins here. On other loci (not reported here) we have noticed subtle differences in binding of MLL between the two cell lines, so physiological differences do exist.

Reviewer #2 (Significance (Required)):

Centromeres function is highly important for genome stability and the epigenetic landscape and the role of transcription in centromere function remains enigmatic. The authors provide several different lines of evidence that the KMT2 methyltransferase family is involved in centromeric transcription and affect loading of centromeric factors (CENP-A, CENP-C, HJURP, and CENP-B). They also show that specific methyltransferases might have both overlapping and unique roles at the centromere. While the reported results for the most part support these conclusions, there are additional experiments needed to backup there conclusions.

Reviewer #3 (Evidence, reproducibility and clarity (Required)):

Transcription at centromere is a rapidly evolving area of research. While initially thought be transcriptionally repressed, it has now been established that centromeric DNA undergo pol II

mediated transcription. Of note, the transcription at centromere is important for centromere assembly and function. Thus, the continuous identification of the factors regulating the centromere transcription and their functional characterization is of substantial interest to centromere biology. Here, Malik and co-authors have found KMT2 family members regulate transcription of human centromeres. The experiments presented in the manuscript show the recruitment of MLL and SETD1A to the centromeres and promote the transcription of alpha-satellite arrays.
The authors go on assess the effects of MLL and SETD1A loss on R-loop formation and RNA pol II occupancy at different alpha-satellite regions. They find while MLL depletion increases R loop formation and pol II occupancy, SETD1A deficiency results in fewer R-loops. They further report that MLL and SETD1A facilities the establishment of centromere by promoting the recruitment of CENP-B, C, HJURP and nascent CENP-A.

Overall, the study is well conducted and nicely written. The transcription and centromere proteins recruitment rescue experiments using MLL-FL and -mutants are elegant. However, there are a few major shortcomings of the study that should be addressed. One is that the study provides multiple ways as to how MLL and SETD1A might be involved in centromere and kinetochore maintenance (R loop formation, centromeric protein recruitment, nascent CENP-A regulation) but none have been explored sufficient detail and as the manuscript stands, most of the results are observational lacking in depth mechanistic insight. Another major issues is the degree to which the transcriptional changes on gene expression induced by MLL and SetD1A can be separated from the direct effects of these genes at centromeric repeats. The other concerns are as follows:

Major
1. The contrasting effects of MLL and STED1A on R-loop and RNA pol recruitment is not adequately addressed. Given both MLL and SETD1A loss produce similar transcriptional effects, it is important to reconcile the disparate effects on R loop formation and RNA pol II (phospho) resulted from MLL or SETD1A loss. Also, can changes in R-loop formation be nullified when combinatorial KD of MLL and SETD1A is used?

We have performed two experiments (as suggested above) which provide some insight into this matter. We have added new data, which shows that (i) combinatorial KD of MLL and SETD1A still produces R-loops accumulation similar to MLL KD alone indicating that these are specific to loss of MLL (Figure 4A); (ii) Although we could not generate KOs of SETD1A, we used shRNA-mediated KD of SETD1A to gauge the RNA Pol II levels (Figure 4F-G). Both total RNA Pol II and RNA Pol II^{S2P} levels are reduced on the centromeres upon SETD1A KD.

Therefore, our data indicates that the disparate effects in R-loop accumulation are most likely due to how MLL and SETD1A may interact differently with the RNA Pol II during (centromeric) transcription. However, as the Reviewer will appreciate, fully resolving the reason for contrasting effects of MLL and STED1A on R-loop and RNA Pol II recruitment, will require extensive experimentation, which we feel is beyond the scope of this manuscript.

(Also see response to Reviewer#1)

2. Does the R-loop formation by MLL loss timing is cell cycle dependent? Does it have any correlation with centromere deposition during the cell cycle?

We thank the Reviewer for this suggestion. To answer this question, based on information provided in recent publication (Kabeche et al., 2018, PMID: 29170278), we performed DRIP experiments in asynchronous cells Vs cells synchronized in mitosis (Figure S4C-D). Our observations indicate that while R-loops do increase in mitosis (Figure S4C), it is unlikely that MLL has a major role in resolving them in this phase (Figure S4C). However, despite repeating the experiment several times, we could not get statistically significant decrease.

In future, we plan to perform DRIP experiments in other cell cycle phases including early G1 phase (after standardizing the duration of CENP-A loading in early G1) in our cells. Hopefully, we will gain insights into the cell cycle dependence of MLL's role in R-loop formation.

3. Is there a direct effect of MLL or SETD1A loss on centromere and kinetochore gene transcripts or protein levels? The authors attribute the dominant negative effect of MLL/SET1D FL on CNEP-C/B recruitment to increased centromere transcription. However, this is distinct from CENP-C/B gene transcription. Author should test and provide the explanation.

We had previously stated in the manuscript that MLL affects CENP-B gene transcription in HEK-293 cells. Further we had provided protein level of CENP-C, B, A and HJURP in MLL iKOs. We have now added protein level of these proteins upon MLL and SETD1A siRNA treatments in U-2OS cells (Figure S6). Further, we have also added quantification from two independent experiments (Figure S6). Our immunoblots and RNA-seq data indicates that the protein level of CENP-C, B, A and HJURP do not change in U-2OS cells but CENP-B shows decrease in MLL iKOs.

Please also see response below on RNA-seq.

4. The current experimental design cannot discount the possibility that transcriptional effects on centromere and/or kinetochore genes (or other unknown factors) upon depletion of MLL or SetD1A by siRNA explain the decrease in CENP-C and CENP-A at centromeres. Likewise, the reduction in alpha-sat may be due to other transcriptional changes up stream of alpha-sat expression. A comprehensive analysis of the transcriptional changes in the MLL and SETD1A transcriptional changes and how they may impact these processes needs to be provided.

It is true that both the proteins being investigated here (MLL and SETD1A) affect transcription of large number of gene-sets and all conclusions should be drawn keeping this fact in mind. Studies outlining these global changes are already published (Mukai et al., 2019, PMID- 31606247; Jin et al., 2018, PMID: 30191958; Wang et al., 2009, PMID: 19703992; Blobel et al., 2009, PMID- 20064463; Milne et al., 2005, PMID-16199523; Guenther et al., 2005, PMID- 15941828; Schraets et al., 2003, PMID- 12789274). Further, transcription of alpha-satellite is an evolving field and all the factors/players affecting these processes are not known.
Still, we have analysed the effect of loss of these proteins by performing RNA-seq in U-2OS cell lines. As shown in Figure S5, Table 1-3, MLL and SETD1A regulate signal transduction, cell adhesion and extracellular matrix organization in U-2OS cells. 3% genes with GO term 'cell cycle' and less than 1% genes with GO term 'RNA pol II transcription' were found to be differentially expressed in MLL-KD and SETD1A-KD. MLL did not show significant perturbation in any centromere genes while upon SETD1A-KD, CENP-H showed downregulation, which is discussed in the manuscript.

In the manuscript we have told the readers about large scale impact of MLL and SETD1A on transcription and mentioned previous effects of these proteins on cell proliferation, including those

from our laboratory. We trust the readers will be able to make informed conclusions keeping all these factors in mind.

5. It is also surprising that CENP-B and CENP-C and CENP-A intensity goes up upon FL-MLL expression. ¬¬¬ Is there more available CENP protein (and RNA under these conditions). Again, knowing the transcriptional changes is critical to understand if there is a direct role for MLL in centromere assembly and function.

We do not know the answer to this question but we speculate that it is the availability of cenRNA which causes the intensity of CENP-A, B and C to go up on the centromere. To support this arguments (i) our RNA-seq experiments indicate that MLL and SETD1A do not regulate CENP-A, B and C transcriptionally (Figure S5); (ii) Our WB analysis indicates that cellular levels CENP-A, B and C remain same upon MLL and SETD1A KD (Figure S6);(iii) of our experiments with SNAP-tag CENP-A (Figure 7, S7) indicate that even when equal or more CENP-A was available, it could not be incorporated when MLL or SETD1A were depleted.

Therefore, we feel that it is increase in cenRNA transcription (Figure 1), which can explain the increase in centromeric intensity of these proteins.

6. Authors should stain the MLL KD+FL/mutant cells with H3K4 Me-2 where HJURP and CENP-A recruitment and nascent CENP-A are examined. This is an important validation to conclude that the CENP-A recruitment impairment is due to defective methylation.

We thank the Reviewer for this suggestion. We have performed IF co-staining on chromatin fibres for H3K4me2 and CENP-A upon MLL or SETD1A KD in U-2OS cells (Figure 7G) as well as cell expressing SET domain mutants (Figure S7 F-G) of these proteins. We observe a drastic reduction in both H3K4me2 and CENP-A on these fibres indicating that indeed the CENP-A recruitment impairment is due to defective methylation.

Surprisingly, the control siRNA + delta SET domain mutant results in contrasting outcome for CENP-A recruitment vs nascent CENP-A in Fig 6 E and F, authors please comment. Also see minor comment 6 about p values.

We are unable to appreciate the Reviewer's concern. The CENP-A recruitment in all cases is decreasing. If the Reviewer is concerned about the levels of CENP-A in Controls of different cell lines, please see our response to Reviewer#2, point 7.

7. The experiments in figure 2A require controls to ensure that signal is specific to MLL, including no primary controls, and conducting the experiment under MLL siRNA conditions.

The siRNA KD to check antibody specificity is shown in Figure S2C-D. We have now added no primary control (Figure S2E).

8. It is somewhat surprising that MLL is restricted to CENP-A localization, as CENP-A occupies only a small portion of the alpha-satellite DNA, and the alpha-sat DNA extends beyond the limited CENP-A domain, as indicated by CENP-B staining. In addition, the authors show that MLL regulates transcription pericentric repeats in figure 1, but the localization of MLL does not reflect this.

We respectfully disagree with the Reviewer in this regard. The overlap between MLL and CENP-A is considerable but not 100%. In fact, when calculated, the Pearson correlation coefficient lies between 0.4 to 0.5 (now added to the legend of Figure 2).

When we looked at the 3D view of our images we observe that MLL/SETD1A show about 50% overlap with CENP-A (Figure S2B). This agrees with Pearson correlation coefficient obtained after quantifying the localization of these proteins as suggested by this Reviewer (see below).

We also wish to clarify that we have not shown that MLL regulates transcription of pericentric repeats. As the α-satellite DNA sequences are also known to be present in peri-centromeres, we wished to ensured that centromeric transcription is addressed by interrogating chromosome 17 specific α-satellite arrays, which have been characterized extensively.

9. The authors show that MLL3 also influences alpha-sat transcription (Figure 1A), but is not analysed further. Is MLL3 also recruited to centromeres and does disruption leads to loss of centromere proteins and CENP-A loading? This is important to understand if MLL is unique or if this is a shared function between MLL complexes.

We hypothesized that this would be a redundant function between MLL complexes as KD of different members decreases cenRNA transcript (Figure 1A) and they are all colocalizing with CENP-A (Figure S2F).
We have performed the KD of MLL3 using siRNA and quantified the centromeric intensity of CENP-A and HJURP (see Reviewer Figure 1 B-C). As shown both CENP-A and HJURP are showing decrease on centromere upon MLL3 KD. However, as the results are still preliminary, we do not wish to include them in this manuscript.

Minor

1. The transcription data and the ChIP presented in Fig1 and -2 respectively should include the control siRNA also and instead of fold change the average of absolute values with all replicate data points should be shown on the bar blot.

We have shown all replicate data points in all plots through-out the manuscript including those presented in Figure 1 and 2 now. Figure 2 plots already show absolute values as percentage of Input. For Figure 1, we will be unable to show absolute values as we did not generate standard curves for all the replicates/experiments. We have used the $2^{-\Delta\Delta C_T}$ method to analyse the relative changes in gene expression (Livak and Schmittgen, 2001, PMID: 11846609) between our Test and Controls. Further, the Fold Change makes the graph easier to read and compare between mutants.

2. The labelling of cell line (samples) is not uniform throughout the manuscript. For instance, fig 1D- it the U-2OS sample should be renamed as parental or vector (whatever is appropriate) as for other samples FL or mutant is used. Please correct elsewhere also in the manuscript.

We have made this correction and indicated U-2OS as parent cell line.

3. Fig 2A: quantification needed for IF.

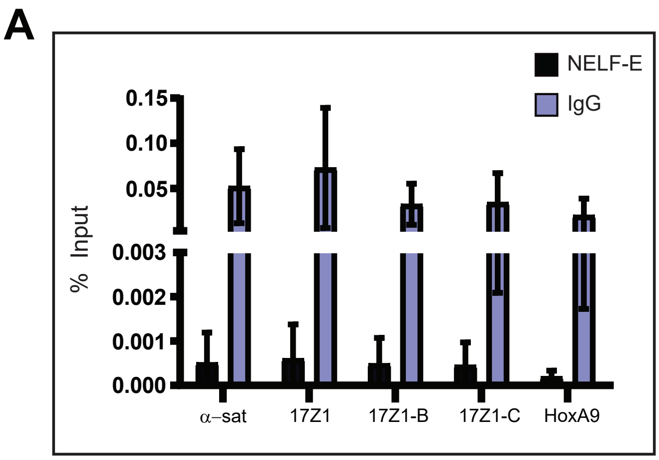

Figure 1A: Chromatin immunoprecipitation with NELF-E antibody.

(A) Chromatin immunoprecipitation with NELF-E and IgG antibodies were performed on HEK-293 cells. Immunoprecipitated DNA was quantified with RT-qPCR and results plotted as percent input enrichment, are shown. ChIP experiment was performed at least twice. Error bars represent SD. α-sat, α-satellite.

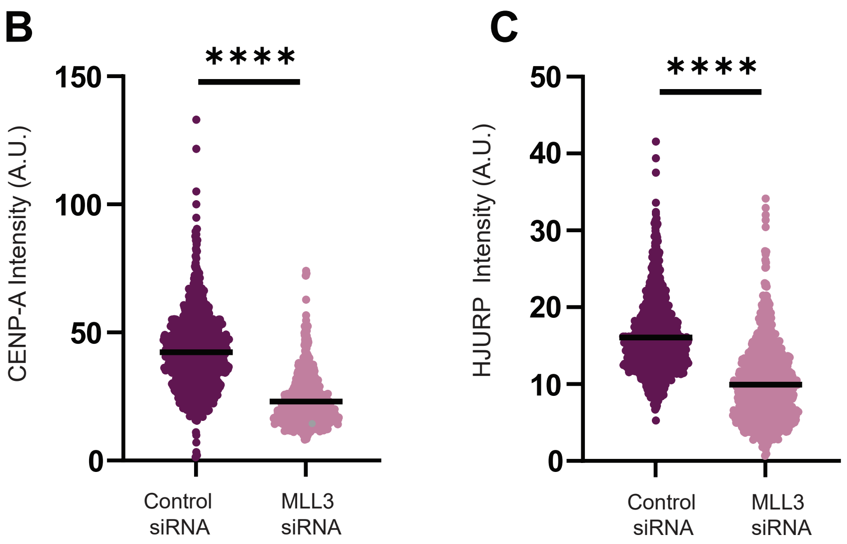

Figure 1B-C: CENP-A and HJURP centromeric protein levels upon MLL3 knock down.

(B,C) Immunofluorescence quantification of CENP-A (B) and HJURP (C) in Control or MLL3 siRNA-treated U-2OS cells. Fluorescence intensities are plotted. Each data point represents a single centromere. The error bar represents SEM. n ≥300 centromeres (n=2experiments). For quantification, Z-stack images were merged and individual CENP-A and HJURP signal intensities were measured using ZEN software. ****P ≤ 0.0001, ns: not significant P > 0.05 (Mann-Whitney two-tailed unpaired test). A.U., arbitrary units.

It is now included in the legend.

4. In all the bar plots, values for each biological replicate for each sample should be included.

We have made this correction.

5. What is C and T in Fig 5C-F, 6E-F, 6I, 6J? If it is "control and treated", it's redundant. Please consider removing it accordingly.

We have made this correction.

6. As many combination are used in figure 5 and 6, a table of p value for all the possible will be helpful to discern the statistical difference between different conditions.

The combination we wish to highlight is between Control and Test from a particular cell line, and p value for these have been indicated in the figures. Despite our best efforts, some variation between Control of different cell lines will exist and may lead to over-interpretation of data. Therefore, we do not wish to confuse the reader by providing the p values for all possibilities.

7. It would be helpful if the authors would adhere to either the MLL or KMT2 nomenclature.

We have changed KMT2 family to MLL family.

Reviewer #3 (Significance (Required)):

If this study is accurate it will have a significant impact on understanding how centromeres function. It will be the first description of transcriptional regulation of centromeric repeats.

Reviewer #4 (Evidence, reproducibility and clarity (Required)):

The authors found KMT2 H3K4me2 methylase family proteins, MLL, MLL2, MLL3, SETD1A, SETD1B as endogenous enzymes involved in the transcription of alpha-satellite DNA, and also found these KMT2 family proteins are localized on metaphase centromere. Of these, MLL depletion reduced the levels of H3K4me2 and H3K9ac modifications in alpha satellite DNA, as well as the centromere proteins CENP-A, CENP-B, CENP-C, and HJURP assemblies. The reduction of centromere proteins assembly was also confirmed under the SETD1A depleted condition. In addition, ChIP enrichment of alpha satellite DNA with S9.6 antibodies increased under MLL depleted conditions, but decreased under SETD1B depleted conditions. This functional contrast between MLL and SETD1A is interesting. However, since depletion of either protein reduces the centromere protein assembly, there might be no link between the functional contrast above and the centromere proteins assembly. Overall, the author investigated several properties of MLL or SETD1A depleted cells and each experiment was performed with a certain quality, but this manuscript does not seem to focus on the conclusion of the title.

Major comments:

Figure 1A and 6: Are the KMT2 family proteins localized to the centromere in the G1 phase as

well as metaphase? If not localized in G1, how are MLL and SETD1A involved in CENP-A deposition?

The Reviewer probably meant Figure 2A and 6. We have checked MLL and SETD1A in early G1 by IF and these images are now included in the manuscript (Figure 2B) along with interphase (Figure S2A) staining.

Is the H3K4me2 modification to be involved the centromere proteins assembly? I could not find results demonstrating that "KMT2 family members regulate H3K4 methylation to ensures kinetochore activity at human centromeres".

We thank the Reviewer for their comment. We have demonstrated using ChIP in MLL iKOs that upon loss of MLL, H3K4me2 (Figure 3C, S3B) is reduced. We also demonstrate that in the same iKO cells at the same loci by N-ChIP that CENP-A and CENP-C recruitment to alpha-satellite DNA (Figure 7E-F) is reduced. Therefore, it is our claim that H3K4me2 modification is involved in the centromere proteins assembly.

However, in order to strengthen this claim, we have performed IF for H3K4me2 and CENP-A on stretched chromatin fibres in presence and absence of MLL or SETD1A (Figure 7G) and SET mutants of these proteins (Figure S7F-G). These experiments show that the H3K4me2 mark decreases upon KD of our HMTs along with corresponding decrease in chromatin bound CENP-A.

We have now changed the title of the manuscript to “KMT2 family members regulate H3K4 methylation to ensure CENP-A assembly at human centromeres”.

Similarly, is there any result indicating a functional link between the MLL or SETD1A dependent centromere proteins assembly and the other MLL or SETD1A related functions such as the alpha satellite DNA transcription, D-loop formation/resolution, and histone modifications.

Several groups working on centromere have established the role of alpha satellite transcription and histone modification to centromere proteins assembly. Our results here add to this research by revealing the identity of HMTs, which deposit H3K4me2 to facilitate alpha satellite transcription. Our systematic KD of each HMT member reveals that all members of MLL family are involved in this transcription process. We study one/two of these HMTs in detail to functionally characterize their role at the centromere.

We have used centromere specific loci from chromosome 17 to show that the same loci which are enriched for MLL binding, are attenuated for transcription, H3K4me2 and associated histone modifications, have decrease in CENP-A and CENP-C proteins upon loss of MLL (Figure 1, 2, 3 and 7E-F). As CENP-A nucleosomes are retained at same HORs/sequences in cycling cells (Nechemia-Arbely et al., 2019, PMID:31160708), our use of specific chromosome 17 loci over general alpha-satellite DNA provides us the flexibility of interrogating the MLL specific functions from different assays and co-relating them to MLL dependent centromere proteins assembly.

As stated in previous response, we agree with the Reviewer that the most relevant functional link here is the H3K4me2 deposition by our HMT with CENP-A assembly and we have performed additional experiments with chromatin fibres to show a co-staining between H3K4me2 and CENP-A in presence and absence of MLL or SETD1A (Figure 7G). Further, the SET domain mutant of MLL

or SETD1A is unable to rescue the loss of H3K4me2 and CENP-A on the chromatin fibres (Figure S7F-G).

Minor comments

Figure 5A: In what cell cycle are the presented cells?
The cells are in mitosis (pro-metaphase).

Figure S2D: MLL shRNA is displayed with light purple bars and a black index. The colours do not match.
We have made this correction.

Figure S2G: Does CENP-B bind to the HoxA9 or CD4 locus? These ChIP recovery rate are higher than alpha sat.

We have included the readings in this plot. As can be seen, alpha-satellite shows reproducible ChIP readings while there is a large variation seen in the readings of HoxA9 and CD4, rendering the binding non-significant statistically.

Reviewer #4 (Significance (Required):

The manuscript includes new information about KMT2 family proteins localization to mitotic centromere and involvement of MLL or SETD1A protein in the transcription, histone modification, and centromere proteins assembly on the human alpha satellite DNA. Therefore this manuscript may be interesting to groups working on centromere research. However, the writing and data sets are not well focused on the conclusions of the title, therefore it may appear descriptive or distracted to the general reader.

We are thankful to all the Reviewers for their critical comments and appreciate their efforts in improving this manuscript.
