# Supplementary material for: MLL methyltransferases regulate H3K4 methylation to ensure CENP-A assembly at human centromeres
Source: PLoS Biol. 2023 Jun 28;21(6):e3002161. doi: 10.1371/journal.pbio.3002161 (PMC10335677; doi:10.1371/journal.pbio.3002161)
Supplement: Attachment — Submitted filename: 126050_1_rebuttal_2388117_rdnql9.docx [file pbio.3002161.s015.docx]

**Manuscript number:** #RC-2022-01403

**Corresponding author(s):** Shweta Tyagi

*[The “revision plan” should delineate the revisions that authors intend to carry out in response to the points raised by the referees. It also provides the authors with the opportunity to explain their view of the paper and of the referee reports.*

*The document is important for the editors of affiliate journals when they make a first decision on the transferred manuscript. It will also be useful to readers of the reprint and help them to obtain a balanced view of the paper.*

*If you wish to submit a full revision, please use our "*[*Full Revision*](https://reviewcommons.org/templates/full-revision.docx)*" template.* ***It is important to use the appropriate template to clearly inform the editors of your intentions.****]*

## 1. General Statements [optional]

*This section is optional. Insert here any general statements you wish to make about the goal of the study or about the reviews.*

We thank all the reviewers for their positive reviews. We note that in general, all reviewers are happy with the quality of the experiments and conclusions drawn. In addition, they would like to see additional experiments done. Some of the key experiments we propose to address common concerns are as follows:

1. Double KD of MLL and SETD1A to assess for synergistic/contrasting affects.
2. Kinetochore fiber co-staining between H3K4me2 and CENP-A in presence and absence of MLL to support our present experiments.
3. Additional experiments on R-loops and Pol II recruitment to provide better clarity on the disparity observed between MLL and SETD1A loss.

The results from above experiments will not only strengthen our present claim but also provide new information relevant to our story. Details of other planned/already performed experiments to address their remaining comments are given in the document.

## 2. Description of the planned revisions

*Insert here a point-by-point reply that explains what revisions, additional experimentations and analyses are planned to address the points raised by the referees.*

**Reviewer #1**

****Major comments:**** 

**I think the experiments within the manuscript are generally of good quality and well controlled. However, I find that the presentation and description of the results were at times confusing. In fact, to get a better grip on the data, I made summary tables to get a better sense of what the data was telling. In my opinion, this manuscript contains findings that are of interest to the field, even when the results might now be clear cut. Personally, I would encourage the authors to consider a follow up where combinations of KMT2 family members are KD to hopefully get a cleaner picture of how nucleosome methylation is regulated at the centromere and how this regulates centromere identity.**

We thank the Reviewer for their comments. With this review process, and suggestions from all four Reviewers, we hope that the manuscript is adequately presented with clear description of the results.

We have taken Reviewer’s suggestion and planned experiments where MLL and SETD1A will be KD and we will assess the cells for i) CENPA levels, ii) R-loop formation.

**+ Did KD of all the KMT2 family members also result in noticeable reduction in protein levels?**

We only checked the protein levels of MLL and SETD1A by IF and WB after siRNA KD. We can also check protein levels of MLL2 and SETD1B. (MLL3 is a large protein (5000 aa) and does not get processed. In the past, we have not been successfully in obtaining MLL3 band on WB.)

**+** **Visual inspection of western blots do not appear to be consistent.**

**Similarly, HA-CENP-A levels are up in Fig S5D, G, H. This appears to be contradictory to the premise of this manuscript that H3Kme2 is essential for guaranteeing centromeric transcription, which in turn is essential for new CENP-A loading. Therefore, the prediction would be that KD of KMT2s would result in loss of H3K4me2 (Fig3C), loss of centromeric transcription (Fig1A) and subsequent loss of new CENP-A loading (Fig 6K,L) as well as total CENP-A (replication-loss is not replenished). Here the authors show by western blot that MLL KD results in increased CENP-A levels (Fig S5D,G,H). As the authors looked at WCE, it is possible that non-chromatinized CENP-A is being detected. It might be worth repeating these western blots for chromatin bound CENP-A only to get a cleaner picture. Additionally, all western blots should be quantified.** 

Our reason for looking at WCE for levels of CENP-A (and other proteins) is to check if protein levels are affected due to transcriptional role of MLL (i.e. any of these proteins are transcriptional targets of MLL; please also see comment 3 by Reviewer #3 and our response). Therefore, the idea is to check for non-chromatinized levels of CENP-A. Chromatin bound CENP-A levels are quantified in the IF images, which show a decrease upon MLL and SETD1A KD (Figure 6).

Additionally, we are planning to perform experiments with kinetochore fibres, in presence and absence of MLL, to show a co-staining between H3K4me2 and CENP-A (in response to comment by Reviewer #4). These experiments will also clarify the doubts of this Reviewer with respect to CENP-A levels on chromatin.

In Figure S5 G,H, the CENP-A is recombinantly expressed from CENP-A-SNAP-3xHA construct with a CMV promoter. This exogenous expression system should not be affected by MLL KD. While we did not expect to see reduction in CENP-A levels in these cells, we did not expect an increase also. However, despite this increase in recombinant CENP-A WCE levels, we observe a decrease in our IF quantification of chromatin CENP-A upon MLL and SETD1A KD (Figure S5E, F).

We will quantify all WB during revision.

**Reviewer #2**

****Major comments:****

**+. H3K9me3 changes do not always correlate with H3K4me2 decrease (not all loci increase when H3K4me2 decreases). Could you please comment on this? Also, there is a massive difference between iKO#11 and #20 when looking at H3K9me3 levels. In figure S3D, it is very difficult to interpret the differences due to the split bar.**

We agree with the Reviewer on this. Even though the final outcome is similar, there seems to be a wide variation in the reading of iKO#11 and iKO#20. We plan to perform additional ChIP experiments with a new batch of H3K9me3 antibody in both clones at the same time and then comment.

**+. I'm confused about the conclusions shown in figure 4A. Here, siRNA of SetD1A is compared to siRNA of SetD1A + Triptolide and show no significant difference. This is used to argue that the changes in R-loop formation is mainly transcription based. However, if one compares the 2 control lanes, 1 and 4, is noticeable that they are almost identical and predictably not significantly different. Does this mean that addition of triptolide and consequently inhibition of transcription has no effect on R-loop formation in their experiment.? The authors should explain this. In addition, it also looks like there a reproducible difference between SetD1A siRNA alone and with triptolide. This should be discussed, too.**

We thank the Reviewer for noticing this discrepancy. According to our hypothesis, the Control and Control + Triptolide should be different. We have checked our other data sets and there is a decrease in Control + Triptolide sample with respect to Control. However, for some reason, the data sets displayed here do not reflect that.

We are planning to investigate the status of R-loops in double KD for MLL and SETD1A (see comments by Reviewer#1, comment 1). To be able to compare samples with each other, we will be acquiring the images from different samples at the same time. We hope that this new data set will take care of this issue. SETD1A siRNA and SETD1A siRNA + Triptolide shows no significant change statistically as indicated in the Figure 4A.

+. In figure 5C, MLL-FL control sample shows reduced amounts of CENP-C compared to both the MLL siRNA treated cells and the rest of the samples. In addition, all control samples expressing MLL seem to have a general reduction in CENP-C signals compared to U2OS cells. This seems to indicate that expression of MLL might rather decrease the amount of centromeric CENP-C.

Similarly, in figure 5D, expression of SETD1A leads to an increase of CENP-C signal, even in the case of SETD1AΔSET. These results indicate that SETD1A itself and not its methyltransferase activity contribute to CENP-C recruitment, which is partially in contradiction with the results shown before. Could you please comment on this?

Please see explanation for next point.

+. Figure 6C, MLL-FL cells show reduced H JURP signal in the control compared to U2OS cells. Has the protein expression of these constructs been assessed (same applies to the mutants and SETD1A)? There is a chance that these unexpected differences might come from expression differences.

As we have stated in the manuscript, we have had problems staining for centromeric proteins in our mutant cell lines, particularly MLL recombinant constructs. As a result, some of these experiments were not done together, introducing variation in the staining (Jonkman et al., 2020, PMID: 32235926). We plan to stain and image the Controls from some of these proteins (say CENP-C and HJURP) at the same time to see if that is the reason for the observed difference.

Further, the Reviewer’s suggestion of assessing the expression levels of the recombinant proteins can also provide an explanation for these differences. However, in our past experience, this has proved very difficult as we had purposefully selected cell lines which showed expression similar to cellular levels. Therefore, we propose to assess either protein or **transcript** level of recombinant proteins in our cell lines.

**+. In the discussion, the increase in S2ph in MLL knockout cells is used to suggest an aberrantly paused RNA polymerase which would lead to reduced centromeric transcription. While this is possible, MLL mutants are known to accumulate RNA pol II at transcription termination sites, and since the authors show that markers associated with active transcription (S2P and H3K36me2) are accumulated in their MLL iKO lines, additional evidence is required to support a paused polymerase. Since RNA polymerase S5ph is generally considered a marker for paused polymerases, especially close to promoters, where H3K4me2 is mostly deposited, I would suggest testing enrichment of S5P and some known RNA polymerase pausing factors (e.g. NELF, DSIF) at the centromeres either by IF or ChIP in the MLL knockdown lines.**

As the status of S5P on the centromeres is not clear (Chan et al., 2012, PMID: 22308327), we plan to use NELF to validate paused RNA polymerase at the centromeres either by IF or ChIP in the MLL iKO cell lines. We thank the Reviewer for this suggestion.

***Minor points:***

**+. I would suggest quantifying the knockdown efficiencies and indicate these underneath the western blot panels.**

We plan to do this during revision.

**Reviewer #3**

****Major comments:****

**+. The contrasting effects of MLL and STED1A on R-loop and RNA pol recruitment is not adequately addressed. Given both MLL and SETD1A loss produce similar transcriptional effects, it is important to reconcile the disparate effects on R loop formation and RNA pol II (phospho) resulted from MLL or SETD1A loss. Also, can changes in R-loop formation be nullified when combinatorial KD of MLL and SETD1A is used?**

While we may not be able to fully resolve the reason for contrasting effects of MLL and STED1A on R-loop and RNA Pol II recruitment in this manuscript, we have planned two experiments (as suggested above). We plan to use NELF to validate paused RNA polymerase at the centromeres either by IF or ChIP in the MLL iKO cell lines.

We have also planned double KD of MLL and SETD1A to gauge for R-loop formation in these double KD cells as suggested here and other Reviewers.

**+. Does the R-loop formation by MLL loss timing is cell cycle dependent? Does it have any correlation with centromere deposition during the cell cycle?**

We thank the Reviewer for this suggestion. We will plan experiments to check if R-loop formation correlates with centromere deposition during the cell cycle.

**+. It is also surprising that CENP-B and CENP-C and CENP-A intensity goes up upon FL-MLL expression. ¬¬¬ Is there more available CENP protein (and RNA under these conditions). Again, knowing the transcriptional changes is critical to understand if there is a direct role for MLL in centromere assembly and function.**

Please see our response for Reviewer#2, point 7 & 8. Hopefully after performing the listed experiments, we can provide an informed opinion to this question.

**+. Authors should stain the MLL KD+FL/mutant cells with H3K4 Me-2 where HJURP and CENP-A recruitment and nascent CENP-A are examined. This is an important validation to conclude that the CENP-A recruitment impairment is due to defective methylation. Surprisingly, the control siRNA +** **delta SET domain mutant results in contrasting outcome for CENP-A recruitment vs nascent CENP-A in Fig 6 E and F, authors please comment. Also see minor comment 6 about p values.**

We agree with the Reviewer and plan to perform co-staining for H3K4me2 and CENP-A (also see explanation for Reviewer #4, point 2).

The CENP-A recruitment in all cases is decreasing. If the Reviewer is concerned about the levels of CENP-A in different cell lines, we hope to address it after performing experiments outlined in response to Reviewer#2, point 7 & 8.

**+. It is somewhat surprising that MLL is restricted to CENP-A localization, as CENP-A occupies only a small portion of the alpha-satellite DNA, and the alpha-sat DNA extends beyond the limited CENP-A domain, as indicated by CENP-B staining. In addition, the authors show that MLL regulates transcription pericentric repeats in figure 1, but the localization of MLL does not reflect this.**

We respectfully disagree with the Reviewer in this regard. The overlap between MLL and CENP-A is considerable but not 100%. In fact, when calculated, the Pearson correlation coefficient lies between 0.4 to 0.5.

We plan to perform Structured Illumination Microscopy (SIM), which can show the extent of overlap between MLL and CENP-A clearly.

**+. The authors show that MLL3 also influences alpha-sat transcription (Figure 1A), but is not analyzed further. Is MLL3 also recruited to centromeres and does disruption leads to loss of centromere proteins and CENP-A loading? This is important to understand if MLL is unique or if this is a shared function between MLL complexes.**

Our guess would be that it is a redundant function between MLL complexes as KD of different members decreases cenRNA transcript (Figure 1A) and they are all colocalizing with CENP-A (Figure S2B).

Based on this suggestion, we plan to analyse the effect of MLL3 on CENP-A loading but we do not plan to explore the binding etc. of MLL3 further.

***Minor points:***

**+. As many combinations are used in figure 5 and 6, a table of p value for all the possible will be helpful to discern the statistical difference between different conditions.**

The combination we wish to highlight is between Control and Test from a particular cell line, and p value for these have been indicated in the figures. As, at present, we do not have explanation for differences between certain cell lines, we do not wish to confuse the reader by providing the p values for these.

In case after analysing the results from the proposed experiments (Reviewer#2, point 7 & 8), we have a better understanding of the differences observed, we will add this table to the Supplementary Information with the explanation.

**Reviewer #4**

****Major comments:****

**+. Is the H3K4me2 modification to be involved the centromere proteins assembly? I could not find results demonstrating that "KMT2 family members regulate H3K4 methylation to ensures kinetochore activity at human centromeres".**

We thank the Reviewer for their comment. We have demonstrated using ChIP in MLL iKOs that upon loss of MLL, H3K4me2 (Figure 3C, S3B) is reduced. We also demonstrate that in the same iKO cells by N-ChIP that CENP-A and CENP-C recruitment to alpha-satellite DNA (Figure 6K-L) is reduced. Therefore, it is our claim that H3K4me2 modification is involved in the centromere proteins assembly.

However, in order to strengthen this claim, we are also planning to perform additional experiments with kinetochore fibres to show a co-staining between H3K4me2 and CENP-A in presence and absence of MLL.

We have now changed the title of the manuscript to “KMT2 family members regulate H3K4 methylation to ensure CENP-A assembly at human centromeres”.

**+. Similarly, is there any result indicating a functional link between the MLL or SETD1A dependent centromere proteins assembly and the other MLL or SETD1A related functions such as the alpha satellite DNA transcription, D-loop formation/resolution, and histone modifications.** 

Several groups working on centromere have established the role of alpha satellite transcription and histone modification to centromere proteins assembly. Our results here add to this research by revealing the identity of HMTs, which deposit H3K4me2 to facilitate alpha satellite transcription. Our systematic KD of each HMT member reveals that all members of MLL family are involved in this transcription process. We study one/two of these HMTs in detail to functionally characterize their role at the centromere.

We have used centromere specific loci from chromosome 17 to show that the same loci which are attenuated for transcription, bearing H3K4me2 and associated histone modifications, have decrease in CENP-A and CENP-C proteins upon loss of MLL (Figure 1, 3, 4 and 6K-L). As CENP-A nucleosomes are retained at same HORs/sequences in cycling cells (Nechemia-Arbely et al., 2019, PMID:31160708), our use of specific chromosome 17 loci over general alpha-satellite DNA provides us the flexibility of interrogating the MLL specific functions from different assays and co-relating them to MLL dependent centromere proteins assembly.

As stated in previous response, we agree with the Reviewer that the most relevant functional link here is the H3K4me2 deposition by our HMT with CENP-A assembly and we are planning to perform additional experiments with kinetochore fibres to show a co-staining between H3K4me2 and CENP-A in presence and absence of MLL.

We are thankful to the Reviewer for their critical comments and appreciate their efforts in improving this manuscript.

## 3. Description of the revisions that have already been incorporated in the transferred manuscript

*Please insert a point-by-point reply describing the revisions that were already carried out and included in the transferred manuscript. If no revisions have been carried out yet, please leave this section empty.*

**Reviewer #1**

****Major comments:****

**+ The title does not reflect the data presented in the manuscript, as kinetochore activity is not tested.**

We received this criticism from other Reviewers also. We have now changed the title to aptly reflect the data presented in the manuscript.

**+ Several cell lines (HEK293, IMR90, and U-2OS) were used to study MLL and SETD1A, but the rational why these cell lines were used and why one cell line was used in one study, but not another is not clearly justified.**

When working with cell lines, in the field, it is generally recommended to try our assays in different cells in order to remove a cell-line specific bias and gauge the function of protein in a general Vs cell-line specific phenomenon. In the same spirit, we have used multiple cell lines. Further, U-2OS cells are generally preferred for IF whereas, HEK-293 are preferred for ChIP/DRIP. IMR90-tert are problematic for both, but as these are non-cancer cells, we used them in our ChIP assays to determine binding of MLL in ‘normal cells’.

Further, even though we tried to generate iKOs in multiple cells, we only achieved success in HEK-293 cells.

Our mutant cell lines were previously made and validated in U-2OS. Hence, we used those for transcript and IF studies.

We have added these statements to the manuscript.

**+ The data is confusing based on previous reports. H3K4me2 is presented as being necessary for centromeric transcription and subsequent maintaining centromere identity. Yet, KD of MLL does not impact RNAP2 levels, increases activated RNAP2, but H3K4me2 levels are significantly down. In contrast, SETD1A KD has a different picture.**

The Reviewer has nicely summed up the conundrum that we wish to highlight. Based on previous and our report, H3K4me2 is indeed necessary for centromeric transcription and subsequent maintaining centromere identity. What we wish to highlight is that two HMTs from the same family, which deposit the H3K4 me2 mark, may differ in other aspects to influence the stability of the centromere differently. This is manifested in the unexpected paused RNA Pol II levels and accumulation of R-loops upon KD of MLL and not SETD1A.

Our findings underscore the fact that both MLL and SETD1A may interact differently with the RNA Pol II during (centromeric) transcription and their loss may have distinct effect on the recruitment/elongation of RNA Pol II. As most pioneering studies on H3K4 HMTs relationship with RNA Pol II were done in yeast (Set1A), how different KMT2 members may influence this relationship in humans is relatively under studied.

**+ The data presented in Figures 5 and 6 were confusing, which is in part due to unclarity of why when what cell line was used. U-2OS is shown as the base-line, as if the addition of MLL-FL, MLLdTAD, MLLdSET are rescue constructs. Why in addition also knock-down MLL. Would this also knock-down the rescue constructs, or only the native proteins? It would be very helpful if these figures are made much easier to interpret.**

We thank the Reviewer for their comment. We have now made corrections by removing U-2OS in Figure 5 and 6. The KD of endogenous wild type transcript is necessary to gauge the effect of mutant proteins alone.

We have explained in the text how our siRNA is directed against 3′ UTR of precursor mRNA for MLL (MLL siRNA #2), which only affects endogenous mRNA. Recombinant cDNA constructs do not have 3’UTR, hence remain unaffected by it.

For SETD1A, we did not have siRNA against 5’ or 3’UTR of precursor mRNA. Therefore, we made recombinant SETD1A constructs, resistant to our siRNA by introducing silent mutations in the region of SETD1A, which will bind the siRNA (Figure S1G, SETD1A siRNA#1) by PCR mutagenesis. As a result, transcripts from all the cDNA constructs of SETD1A used here, will not be affected by SETD1A siRNA#1.

**+ Visual inspection of western blots do not appear to be consistent. MLL KD as shown in Fig S2C is less efficient compared to Fig3.**

We would like to draw the attention of the Reviewer towards the fact that Fig S2C shows transient **knock-down** of MLL using shRNA whereas Fig3A shows levels of MLL in our inducible **knock-out** cells. shRNA treatment-mediated knock down is less efficient and that is also reflected in our blots.

**Reviewer #2**

****Major comments:**** 

**+ While the IF images for centromere localisation of MLL1 and SetD1A in mitotic cells are truly remarkable (Figure 2 and 5, etc), it is important to show an interphase staining for both proteins as well. First of all it would be interesting to see if the centromere staining persists in interphase, but more importantly, a genome wide staining would be expected and provide an important validation of the antibodies.**

We thank the Reviewer for their appreciation. Now we have included interphase (Figure S2D) as well as early G1 (Figure 2B) staining.

**+. In figure S4B, samples are expressed as percentage of input. They are above 100%, even 500% in some cases. This would mean that more DNA than the input DNA is pulled down in the assay, which is not possible. It is likely that input sample Ct values have not been adjusted for the dilution factor in the assay. I would suggest checking these calculations again. If the other samples have been treated in the same way before calculating fold enrichment, they should be checked too.**

We thank the Reviewer for pointing out this lapse. As suggested, we have corrected the values and replotted the graph S4B which now falls in the range as expected (we used similar correction formula to calculate ‘% Input’ as in Sanz and Chedin, 2020, PMID: 31053798). The data in Figures 4B and 4C will remain the same, as they are relative enrichment values compared with control cells.

**+. Fig 4D Hox9 is not statistically significant, while 4E 17Z1 is significant. Is this correct? As mentioned before, it is not clear what the significance has been calculated against, whether against the control or the rest of the loci.**

It is correct. The significance has been calculated against mean of Control Vs mean of Test. However, when we do it against rest of loci, the significance still remains the same as with above method.

***Minor points:***

**+. The paper repeatedly ignores previously published work on centromere transcription in model organism like S. pombe, which spear headed research in centromere transcription (Ekwall and Allshire labs) and in Drosophila on CENP-A stability (Heun lab). These should be cited as centromere transcription is conserved in evolution and does not just exist in human cells.**

We apologize for this omission. We have now included these citations in the manuscript.

**+. Figure S2G. Is there truly enrichment compared to IgG, or is it different between centromeric loci and control genes? It is not clear whether significance is indicated compared to IgG or between centromeric loci or control samples. This applies to most plots. I would mention it either in the legend or indicate in the plot if possible.**

There is enrichment compared to IgG. The significance is calculated against mean of test genes Vs mean of IgG. As it is uniformly applied to all plots, we have now mentioned it in the Methods section.

**+. In the plots containing a break in the Y axis, the unit immediately after the break should be indicated. While the reason for introducing the break is clear given the differences between samples, the lack of that unit hampers correct comparison between samples on the two sides of the break.**

We have corrected the plots and shown units on both sides now.

**+. In all plots with y-axis written as "Fold over Control", I would suggest indicating what is assessed in the axis label or at least in the title (like in figure 3). Having to look for it in the text or in the legend make the figures a bit confusing.**

We have corrected as advised.

**+. Two sets of different siRNAs and shRNAs have been used for MLL1 and SETD1A knockdown. Is there a reason for this change? If so, I would suggest mentioning this in the text.**

We have done most of our studies with siRNAs. For ChIP, however, we needed a greater number of cells. Hence, we used shRNA-transfected cells to obtain KD of our proteins.

We have added this in the text.

**+. It would be better to add the siRNAs sequences in the methods, instead of just referencing them.**

We have added the siRNA sequences in the Supplementary Information.

**+. Immunofluorescence is generally shortened as IF. I would suggest keeping this consistent with known literature.**

We have made this correction.

**+. In Figure S2A, scale bars are different between MLL1 and SETD1A. Since MLL1 siRNA cells seem to have a chromosome de-condensation phenotype or larger cells, it might help to have all images with the same magnification to allow proper comparison.**

The cells are at the same magnification but the scale bar was incorrectly drawn. We thank the Reviewer for noticing this anomaly. It is now corrected.

**+. Why do non transformed cells (IMR-90 tert) have much less enrichment of MLL, SETD1A, H3K4me2? Is the cell line problematic in ChIP assays, or do you think there are physiological differences?**

Probably both factors contribute to the less binding observed for our proteins here. On other loci (not reported here) we have noticed subtle differences in binding of MLL between the two cell lines, so physiological differences do exist.

**Reviewer #3**

****Major comments:****

**+. Is there a direct effect of MLL or SETD1A loss on centromere and kinetochore gene transcripts or protein levels? The authors attribute the dominant negative effect of MLL/SET1D FL on CNEP-C/B recruitment to increased centromere transcription. However, this is distinct from CENP-C/B gene transcription. Author should test and provide the explanation.**

We have tested the protein levels of CENP-A, CENP-B, CENP-C and HJURP in MLL iKOs (Figure S5A-D) and reported that CENP-B protein levels are affected by loss of MLL.

We can perform WBs for the same upon SETD1A KDs.

Further, we have analysed the data from published reports (Jin et al., 2018, PMID: 30191958; Wang et al., 2009, PMID: 19703992) and found that transcripts of CENP-A, CENP-B, CENP-C and HJURP are either unchanged or show non-significant differential gene expression upon loss of MLL (in MEFs) and SETD1A (in MCF-7 cells). With this data, and our own WB experiments, we believe that gene transcription of these proteins is mostly unaffected upon loss of MLL or SETD1A. (Also see below).

| **MLL-/-** |  |  |  |
| --- | --- | --- | --- |
| **Ensembl_GeneId** | **Gene Symbol** | **Fold Change (log2)** | **p adj** |
| ENSG00000115163 | CENP-A | 0.580632624 | 0.250252685 |
| ENSG00000125817 | CENP-B | -0.11070372 | 0.753904197 |
| ENSG00000145241 | CENP-C | 0.755946485 | 0.19969252 |
| ENSG00000123485 | HJURP | 0.910690133 | 0.001502133 |
|  |  |  |  |
|  |  |  |  |
| **shSET1A** |  |  |  |
| **Ensembl_GeneId** | **Gene Symbol** | **Fold Change (log2)** | **p adj** |
| ENSG00000115163 | CENP-A | -1.040346744 | 0.012698429 |
| ENSG00000125817 | CENP-B | -0.004369512 | 0.992969119 |
| ENSG00000145241 | CENP-C | 0.103789684 | 0.834848218 |
| ENSG00000123485 | HJURP | 0.37708933 | 0.190920574 |

**+. The current experimental design cannot discount the possibility that transcriptional effects on centromere and/or kinetochore genes (or other unknown factors) upon depletion of MLL or SetD1A by siRNA explain the decrease in CENP-C and CENP-A at centromeres. Likewise, the reduction in alpha-sat may be due to other transcriptional changes up stream of alpha-sat expression. A comprehensive analysis of the transcriptional changes in the MLL and SETD1A transcriptional changes and how they may impact these processes needs to be provided.**

It is true that both the proteins being investigated here (MLL and SETD1A) affect transcription of large number of gene-sets and all conclusions should be drawn keeping this fact in mind. Studies outlining these global changes are already published (Mukai et al., 2019, PMID- 31606247; Jin et al., 2018, PMID: 30191958; Wang et al., 2009, PMID: 19703992; Blobel et al., 2009, PMID- 20064463; Milne et al., 2005, PMID-16199523; Guenther et al., 2005, PMID- 15941828; Schraets et al., 2003, PMID- 12789274). Further, transcription of alpha-satellite is an evolving field and all the factors/players affecting these processes are not known.

Still, we have analysed the effect of loss of these proteins from data sets mentioned above and Venn diagram representing DGA of gene-sets in GO: term ‘Centromere’ and ‘Mitosis’ are shown for both the proteins for Reviewer’s consideration. As can be seen, the genes related to ‘centromere function’ in the both cases are largely unaffected while ‘mitotic’ genes represent about 5% of total affected genes for MLL and SETD1A.

In the manuscript we have told the readers about large scale impact of MLL and SETD1A on transcription and mentioned previous effects of these proteins on cell proliferation, including those from our laboratory. We trust the readers will be able to make informed conclusions keeping all these factors in mind.

**+. The experiments in figure 2A require controls to ensure that signal is specific to MLL, including no primary controls, and conducting the experiment under MLL siRNA conditions.**

The siRNA KD to check antibody specificity is shown in Figure S2A. We have now added no primary control (Figure S2C).

***Minor points:***

**+. The transcription data and the ChIP presented in Fig1 and -2 respectively should include the control siRNA also and instead of fold change the average of absolute values with all replicate data points should be shown on the bar blot.**

We have shown all replicate data points in all plots including those presented in Figure 1 and 2 now. Figure 2 plots already show absolute values as percentage of Input. For Figure 1, we will be unable to show absolute values as we did not generate standard curves for all the replicates/experiments. We have used the 2^−ΔΔ^*^C^*_T_ method to analyze the relative changes in gene expression (Livak and Schmittgen, 2001, PMID: 11846609) between our Test and Controls.

**+. The labeling of cell line (samples) is not uniform throughout the manuscript. For instance, fig 1D- it the U-2OS sample should be renamed as parental or vector (whatever is appropriate) as for other samples FL or mutant is used. Please correct elsewhere also in the manuscript.**

We have made this correction and indicated U-2OS as parent cell line.

**+. Fig 2A: quantification needed for IF.**

It is now included in the legend.

**+. In all the bar plots, values for each biological replicate for each sample should be included.**

We have made this correction.

**+. What is C and T in Fig 5C-F, 6E-F, 6I, 6J? If it is "control and treated", it's redundant. Please consider removing it accordingly.**

We have made this correction.

**+. It would be helpful if the authors would adhere to either the MLL or KMT2 nomenclature.** 

We have changed KMT2 family to MLL family.

**Reviewer #4**

****Major comments:****

**Figure 1A and 6: Are the KMT2 family proteins localized to the centromere in the G1 phase as well as metaphase? If not localized in G1, how are MLL and SETD1A involved in CENP-A deposition?**

The Reviewer probably meant Figure 2A. We have checked MLL and SETD1A in early G1 by IFS and these images are now included in the manuscript (Figure 2B) along with interphase (Figure S2D) staining.

***Minor points:***

**Figure 5A: In what cell cycle are the presented cells?**

The cells are in mitosis (pro-metaphase).

**Figure S2D: MLL shRNA is displayed with light purple bars and a black index. The colors do not match.**

We have made this correction.

**Figure S2G: Does CENP-B bind to the HoxA9 or CD4 locus? These ChIP recovery rate are higher than alpha sat.**

We have included the readings in this plot. As can be seen, alpha-satellite shows reproducible ChIP readings while there is a large variation seen in the readings of HoxA9 and CD4, rendering the binding non-significant statistically.

## 4. Description of analyses that authors prefer not to carry out

*Please include a point-by-point response explaining why some of the requested data or additional analyses might not be necessary or cannot be provided within the scope of a revision. This can be due to time or resource limitations or in case of disagreement about the necessity of such additional data given the scope of the study. Please leave empty if not applicable.*

**Reviewer #1**

****Major comments:****

**+ The authors frankly state that the antibody S9.6 is not the ideal method to detect DNA:RNA hybrids, so they do DRIP experiments as well. Yet, DRIP still relies on S9.6. It might therefore be worthwhile to consider using catalytically dead RNaseH fused with GFP, as described in Crossley et al JCB 2021.**

While we appreciate the relevance of this opinion, we hope that the Reviewer will agree that this experiment will require considerable time and effort and yet, not add extra value to our current findings. Moreover, previous reports suggest a disparate bias towards these approaches i.e., DRIP and R-ChIP (ChIP with dead RNaseH) in mapping genomic R-loops (Chedin et al 2021, PMID: 33411340). This is the reason we carried out the widely accepted **classical DRIP** experiment by incorporating RNase H as a control, to validate our IF results.

We will surely consider this suggestion in our follow up studies, to understand the contrasting effects of R-loops accumulation in MLL/SET1A depleted cells.

**Reviewer #2**

****Major comments:****

**+ My major concerns with this study are the potential secondary effects when you tamper with transcription of prolonged periods of time in the cells. As the authors state themselves "MLL1 (and SetDB1?) is essential for cell viability and growth" - keeping that in mind the experimental setups for siRNA experiments extend from 48-72h (couldn't find the description for CRISPR in the methods section). In any case, a lot can go wrong over the course of 3 days without proper transcription, so the authors should show evidence that cells are proliferating normally and moving fine through the cell cycle, similar to wildtype. This is also important as for instance loading of CENP-A itself requires moving from mitosis into G1. In general, faster acting ways to deplete proteins, such as degron-based approaches would be more suitable as an experimental setup.**

We thank the Reviewer for their comment. Cas-9 induction is now listed under ‘Generation of Stable Cell Lines’ and goes on for 7 days. **We plan to include FACS data from iKO cells to show that these cells are proliferating.**

Further, all the images shown for CENP-A loading are of cells released in early G1 stage (Figure 6 A-B, H).

We agree with the Reviewer that the time required to efficiently degrade MLL or SETD1A protein using siRNA method is enough to cause problems for the cell. In fact, we have ourselves reported that about 50% cells undergo stable arrest upon MLL siRNA (Ali et al., 2014, PMID: 24880690) after 72hrs and cells which enter mitosis spend long time in pro-metaphase or metaphase (Ali et al., 2017, PMID: 28633016). Taking all these factors into account, we have released cells for 60 mins (mitotic cells) or 90 mins (early G1) after nocodazole arrest and fixed them thereafter. This was to ensure that we catch only proliferating cells.

We have stated/highlighted these limitations in the manuscript Result or Discussion section.

We agree with the Reviewer that the degron-based approach will indeed simplify our experimental set-up. We have been trying to obtain knock-ins for MLL-degrons but so far we have not achieved success. It is our general experience that genome editing experiments involving *MLL*/*SETD1A* (compared to other genes used in our lab) are not easy to accomplish. While it may become a part of our future experimental set-up, at this time, looking at our past failed attempts, we cannot plan to include such experiments in this manuscript. We would like to draw the attention of the Reviewer to the experiments performed in HAC (Bergmann et al., 2011, PMID: 21157429). Even when LSD1 expression results in rapid loss of H3K4me2, 50% decrease was observed in CENP-A after 3 days and segregation defects were observed 7 days later, indicating that loss of CENP-A can be tolerated over several divisions. This indicates that even by using the degron approach if we are able to achieve H3K4me2 depletion rapidly, we may still have to wait 3 days (same time as used here for siRNA treatment) to observe a reduction in centromere proteins.

(Also see responses to Reviewer#3)
