# Supplementary material for: MLL methyltransferases regulate H3K4 methylation to ensure CENP-A assembly at human centromeres
Source: PLoS Biol. 2023 Jun 28;21(6):e3002161. doi: 10.1371/journal.pbio.3002161 (PMC10335677; doi:10.1371/journal.pbio.3002161)
Supplement: Attachment — Submitted filename: Response to Reviewers.docx [file pbio.3002161.s018.docx]

Response to Reviewers:

Reviewer (them/ their)

We thank all the Reviewer for their insightful comments and suggestions.

Reviewer #1: The authors have satisfactorily addressed all concerns raised.

We thank the Reviewer for their comments.


Reviewer #3: This is a revised manuscript from Malik and colleagues examining the role of MLL and SetD1A in centromere specification and transcription. Overall, the revisions have improved the manuscript, and most of the issues that were raised previously have been addressed. I think the weakness of the manuscript is that the data show that MLL and SetD1A influence centromeric transcripts, but the experiments do not demonstrate that the centromeric transcripts are the mechanism by which CENP-A and HJUPR recruitment are altered in MLL and SetD1A knockdown. But I will stipulate that this may be difficult to directly demonstrate, and I do not think they have been sufficiently proven in the literature.

It is very interesting that the data suggest that SetD1A and MLL may be functioning differently, as SetD1A alters CENP-H and CCTT expression, both of which influence CENP-A deposition (see Okada 2006). The manuscript incorrectly states that CENP-H does not influence CENP-A deposition, however, Okada et al 2006 showed otherwise.  The data that show SetD1A + MLL knockdown have a more drastic phenotype is excellent to see in the manuscript and solves of the big issues I had with the original manuscript, which was why do Set1DA and MLL both show phenotypes. However, it should be noted that despite the statement on page 19, "Our observations indicate that both MLL and SETD1A contribute to CENP- A recruitment synergistically." there is no data for synergy, which would require additional analysis.  However, they could very well be additive, which is consistent with altering multiple pathways.

One last point is that the analysis of the transcriptional changes is insufficient to dispel the concern that MLL is altering transcription of upstream regulators of CENP-A deposition.  Besides HJURP, CENP-C and CENP-A there are many other genes that could negatively alter CENP-A deposition. At the very least, the transcriptional effects of SetD1A and MLL should be shown for Mis18a, Mis18b, Mis18BP1, CENP-I and PLK1. There are others suggested in the literature as well, and the authors should make an effort to pick these out of the RT PCR data to show whether they are changed.

We thank the Reviewer for their comments.

We have changed synergistically to additively.

Okada et al. showed that CENP-H depletion affects new CENP-A loading but not the endogenous CENP-A (Okada et al., 2006; PMID: 16622420). We have added this line in the manuscript and discussed these results in context with reports published later on CENP-H’s role in CENP-A deposition.

We have analysed approximately 20 genes for MLL and SETD1A each, 15 common, which are likely to affect CENP-A deposition directly or indirectly and 3-4 unrelated genes, which were shown to be affected in specific data sets (MLL or SETD1A) our RNA-seq analysis. In the 15 common genes we choose transcripts of genes related directly to the centromere like Constitutive Centromere Associated Network (CCAN) proteins [CENP-L, CENP-I, CENP-N, CENP-O, CENP-T] , Mis18α, Mis18β, and Mis18BP1. We also chose some genes which have been implicated in CENP-A deposition in the literature like p53BP1, KDM5A, SSRP1, AURORA-B, ZFAT and PDS5A (Ishikura et al., 2020; PMID 32997115, Kabeche et al., 2018; PMID: 29170278, Huang et al., 2016; PMID: 27052177, Okada et al., 2009; PMID: 19625449, Okada et al., 2006; PMID: 16622420, Fujita et al., 2007; PMID: 17199038, Jullien et al., 2002; PMID: 11801725). Finally we chose 3-4 genes which were shown to be down regulated in MLL (CD180, APOL4, MYL4, MESI1) and SETD1A( PRM1, RPA3, CENP-H) data sets.

The results are presented as Supplementary Table VI. Consistent with our RNA-seq data, this analysis shows no significant change in the RNA transcript level of all 15 of above mentioned transcript( other than CENP-H, which we have reported in our findings previously). The genes showing down regulation in specific data sets (MLL and SETD1A) in our RNA-seq also showed significant reduction in our qRT-PCR experiments indicating that our RNA-seq data truly reflects the transcript status of MLL and SETD1A siRNA treated cells.

Reviewer #4: Major comment

By the addition of control and/or double knockdown experiments in each revised figure, datasets are now comparable and easier to understand than before. In addition, the title was changed to focus solely on functional link between endogenous centromere H3K4me2 transferase and nascent CENP-A assembly mechanism. Data on the involvement of SET domain in the CENP-A assembly mechanism is also added, and supported conclusion of the title. The manuscript had been improved.

Minor comments
Since the double knockdown of MLL and SETD1A synthetically reduced nascent CENP-A assembly (Figure 6H), unique properties of both MLL and SETD1A, such as the different PolII or R-loop regulation manner, might also be involved in nascent CENP-A assembly. If the functional link between the PolII or R-loop regulation and nascent CENP-A assembly is investigated, the Figure 4 will fit the manuscript more tightly.

We thank the Reviewer for their comments. It will certainly be insightful and pertinent to MLL and SETD1A biology to resolve the functional link between the Pol II/R-loop regulation and nascent CENP-A assembly. However, as the Reviewer will agree that this will require an in-depth analysis to answer the question satisfactorily. Therefore we will be addressing it as a separate project in our follow up studies later.

In the Figure 7G, how can the authors identify which fiber is centromere? And, how frequent can the authors observe such the events per centromere fiber? If the authors add the results of fiber imaging, number or frequency of the events should be shown.

We thank the Reviewer for their comments. In the knock-down cells, as CENP-A is diminished, it was indeed hard to pick centromere fibers. Therefore, the fiber was identified as a centromere fiber by increasing the contrast in post image analysis and looking for CENP-A staining. We have added the frequency of occurrence of H3K4me2 in CENP-A centromere fibers in the figure legends.
